# Research on LPV-H_∞_ control strategy of magnetorheological semi-active suspension with air spring

**DOI:** 10.1038/s41598-025-15552-1

**Published:** 2025-11-10

**Authors:** Gang Li, Shaohua Zhang, Jiantao Li, Qingsheng Huang, Qianjie liu, Lin Zhong, Wenjun Sun, Ruqi Ding, Guoliang Hu, Jianming Deng

**Affiliations:** 1 Key Laboratory of Vehicle Intelligent Equipment and Control of Nanchang City, East China Jiaotong University, Nanchang, 330013 China; 2 School of Mechatronics and Vehicle Engineering, East China Jiaotong University, Nanchang, 330013 China; 3 Yingtan Applied Engineering School, Yingtan, 335211 China; 4 Jiangxi Isuzu Motors Co., Ltd, Nanchang, 330100 China

**Keywords:** Magnetorheological damper, Semi-active suspension, Air spring, Improved hyperbolic tangent model, LPV-H_∞_ control, Engineering, Mechanical engineering

## Abstract

In order to improve the ride comfort and safety of the vehicle, a linear parameter varying H_∞_ control of magnetorheological semi-active air suspension was proposed to solve a series of problems caused by parameter changes and external disturbances. Based on the improved hyperbolic tangent model, the forward magnetorheological damper model was established, and the inverse magnetorheological damper model was established by using CNN. The accuracy of the mechanical model of the magnetorheological damper was verified by comparing the tensile test and simulation data. A linear parameter varying H_∞_ controller (LPV-H_∞_) based on quarter semi-active air suspension model was designed to solve the problem of time-varying system caused by the traditional H_∞_ optimal control which cannot observe and estimate the parameter changes in real time. After solving the controller through the LMI toolbox, simulations were carried out using speed bump road surface and random road surface, and real vehicle tests were carried out using continuous trapezoidal speed bumps road surface, bad road surface and Belgian road surface. The results show that the root mean square values of three performance indexes of MR semi-active suspension under LPV-H_∞_ control strategy are better than those of H_∞_ control and passive control. Compared with H_∞_ control, LPV-H_∞_ control increased by 11.60%, 14.26% and 23.12% in Sprung mass acceleration, Suspension working space and Dynamic tire Deformation, respectively. Compared with passive control, it is improved by 22.50%, 33.64% and 17.12%, respectively. LPV-H_∞_ control has a significant improvement in vehicle ride comfort.

## Introduction

The suspension system is one of the key components of the automobile, and it is also an important part of the force transfer between the wheel and the car body. Automobile suspension system is composed of shock absorber, elastic element and guiding device^1^. Its main function is to slow down the external speed bump force suffered by the car during the driving process to ensure the comfort of passengers and the integrity of the goods, and reduce the car roll, has a guiding role, and ensure that the tire can run in accordance with a certain track.

According to whether the damping and stiffness of the suspension change with the driving conditions, the vehicle suspension can be roughly divided into three categories: passive suspension, semi-active suspension and active suspension^2^. Passive suspension has fixed damping and stiffness, which cannot adapt to the vibration caused by body parameters and road surface changes. The active suspension applies active force to the suspension through the actuator^3^. Although it achieves superior suspension performance, it has the disadvantages of complex structure and high energy consumption. The semi-active suspension is better than the traditional passive suspension in damping characteristics, and the vibration reduction performance similar to that of active suspension can be obtained through appropriate control strategies. The semi-active air suspension can control the height of the chassis, the inclination of the body and the damping coefficient of vibration reduction, which significantly improves the driving experience and increases the ride comfort and safety. Magnetorheological (MR) damper is a kind of intelligent material device of controllable fluid damper with excellent performance. It has the advantages of continuous adjustable damping force, low energy consumption, simple structure and rapid response^4,5^. Therefore, MR semi-active suspension with air spring has become a hot research topic in automotive industry in recent years.

Mechanical models of the MR damper are divided into forward and reverse models. According to the relative motion state of the damper piston and the current input of the excitation coil, the forward model can predict the damping force output by the damper, while according to the relative motion state of the damper piston and the expected damping force, the inverse model can predict the current required by the damper excitation coil^6–8^. Therefore, it is crucial to establish a suitable mechanical model for the MR damper. Mechanical models of the MR damper are mainly divided into parametric models and non-parametric models. Bingham model, Bouc-Wen model, improved Sigmoid model, Spencer model, hyperbolic tangential model, etc.^9–11^, have been extensively studied on parameter models, which have been applied to the control of MR semi-active suspension. The non-parametric model includes neural network model, polynomial model, differential equation model, T-S model, etc.^12^, but the non-parametric model only carries out intelligent control in algorithm, and does not really consider the rheological characteristics of magnetorheological fluid in different occasions, so Kwok et al.^13^. A hyperbolic tangent model is proposed, which can describe the mechanical properties of MR damper well. However, due to the large number of parameters to be identified, Guo S et al.^14^. An improved hyperbolic tangent model is established, which has the advantages of fewer identification parameters, less identification complexity and easy improvement of approximation accuracy.

Air spring has low natural frequency and variable elastic stiffness, can better absorb external vibration energy, and light weight, less noise, is widely used in automobile suspension system important components. The natural frequency of the helical spring is negatively related to the load, and the frequency decreases when the load increases, but this characteristic cannot meet the actual needs of the automobile^15^. The natural frequency of the air spring is not affected by the load and remains stable, so that the vehicle always maintains better handling stability and riding comfort, which meets the actual needs. The combination of air spring and MR damper means that the suspension has lower stiffness and controllable damping, which can better improve the vibration isolation performance of the vehicle suspension system.

For semi-active suspension, similar damping performance to active suspension can be obtained by selecting the appropriate control strategy. Teng M designed an optimized fuzzy skyhook controller with grey wolf optimizer (GWO) algorithm base on a new neuro-inverse model of the MR damper^16^. Yongshuai W Comprehensively considered sprung and unsprung mass, and proposed an integrated skyhook-active disturbance rejection control (ADRC) strategy for vibration reduction of automotive semi-active suspension with MR damper^17^. The sky-hook control can reduce the vertical acceleration of the body of the suspension system and improve the ride comfort of the vehicle, but it cannot improve the impact from the ground^18^. Žáček Jiří developed a unique experimental bench, using a fast magnetorheological damper experimental trolley (with a response time of 3µs) and an improved Groundhook algorithm to control the damper^19^. The ground-hook control can achieve the purpose of reducing the vibration of the wheel and improve the impact of the tire, but it sacrifices the ride comfort of the vehicle^20^. Tien N D proposed a PID controller for the active suspension system of electric vehicles, which uses Ziegler-Nichols for parameter tuning, in order to improve ride comfort^21^. Li M considering the secondary excitation caused by wheel motor drive and vehicle-road coupling, a coupled-dynamics model of a semi-active-suspension vehicle-road system for vertical vehicle motion is investigated under multiple excitations, a BP neural network PID controller based on the sparrow search algorithm optimization is proposed for the semi-active-suspension system. The PID control has the advantages of small amount of calculation and good real-time performance, but its parameter tuning method is time-consuming and laborious, and the control effect is poor^22^. Moaaz O A uses magnetorheological damper and fuzzy logic to control their combination, which will enhance the performance of the suspension system^23^. M.S. developed a semi-active suspension system controller to enhance comfort and road holding by applying adaptive fuzzy logic control theory and combining it with Kalman filtering for state estimation^24^. Fuzzy control is good at dealing with highly nonlinear models and has good robustness, but the accuracy of fuzzy control is low and the stability is poor. Adaptive fuzzy control is suitable for nonlinear and multivariable parameter models, but it is not sensitive to parameter and environmental changes^25,26^. Although the hybrid model predictive control has low accuracy and convenient modeling, it is not suitable for nonlinear time-varying uncertain systems^27^.

The coefficient matrix of the linear parameter varying(LPV) system can observe and estimate the dynamic changes of parameters in real time, which is widely used in some fields with wide range of parameter changes and high velocity^28^. D. Leith verified that the robust variable gain control method based on the LPV system is one of the best methods for dealing with nonlinear systems^29^. However, in the actual control system design, due to the uncertainty of the system and external random interference, the designed controller needs to have strong robustness and anti-interference ability^30^. H_∞_ optimal control can just meet this requirement, but H_∞_ optimal control can’t real-time observation and estimation of time-varying systems caused by parameter changes^31^. Therefore, a semi-active suspension with air spring control system based on a quarter MR damper based on linear parameter varying H_∞_ (LPV-H_∞)_ control is established to transform it into a convex optimization problem, and the control gain can change in real time, which not only ensures the closed-loop stability of the system, but also makes the model more accurate, thus improving the control quality.

In this work, this paper aims to demonstrate that the MR semi-active suspension with air spring can achieve excellent comfort and road holding during vehicle operation, and ensure that the suspension has good stability performance when safety requirements are met. Moreover, this paper also designs a controller that can ensure robustness to cope with the real-time observation of the system and the dynamic changes of parameters, which remains a problem to be solved.

The contribution of this paper is twofold:

- In this paper, MR damper is used to achieve semi-active suspension control. Such damper can obtain good comfort, enhance wheel grip and ensure safety performance. An improved hyperbolic tangent forward dynamic model with fewer identification parameters and higher accuracy, and a convolutional neural network reverse dynamic model with strong feature extraction and classification capabilities are established. They can effectively convert the magnetorheological damper into a mathematical model and replace the MR damper in the simulation analysis process. The accuracy of the dynamic model of the MR damper was verified through comparative test and simulation data.
- Based on the vehicle model, the LPV-H_∞_ control was proposed. This control combines the linear variable parameter system with the traditional H_∞_ control. It can not only observe and estimate the dynamic changes of parameters in real time, but also ensure that the system has strong robustness and anti-interference ability. Taking random road surface and special road surface as inputs respectively, simulation comparative analysis was conducted on the magnetorheological semi-active suspension system with air spring to verify the effectiveness of the proposed control strategy in vehicle suspension vibration reduction.

## Mechanical modeling of MR damper

### Mechanical properties test of MR damper

**Fig. 1:**
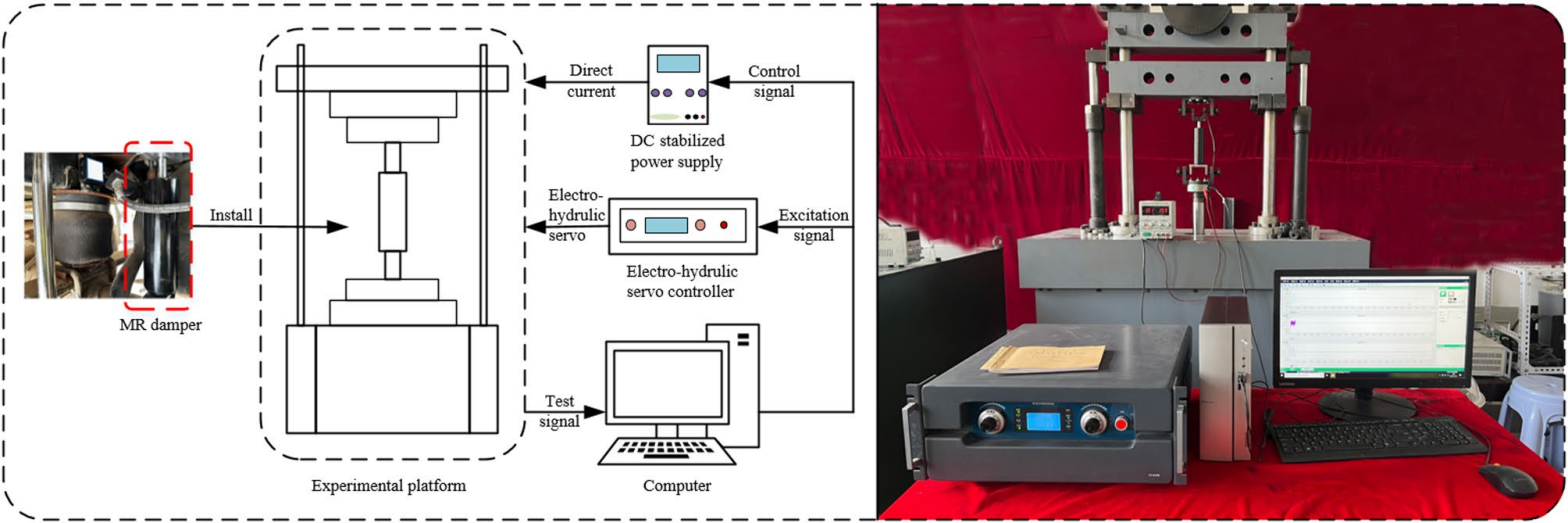
MR damper mechanical properties test system.

**Fig. 2:**
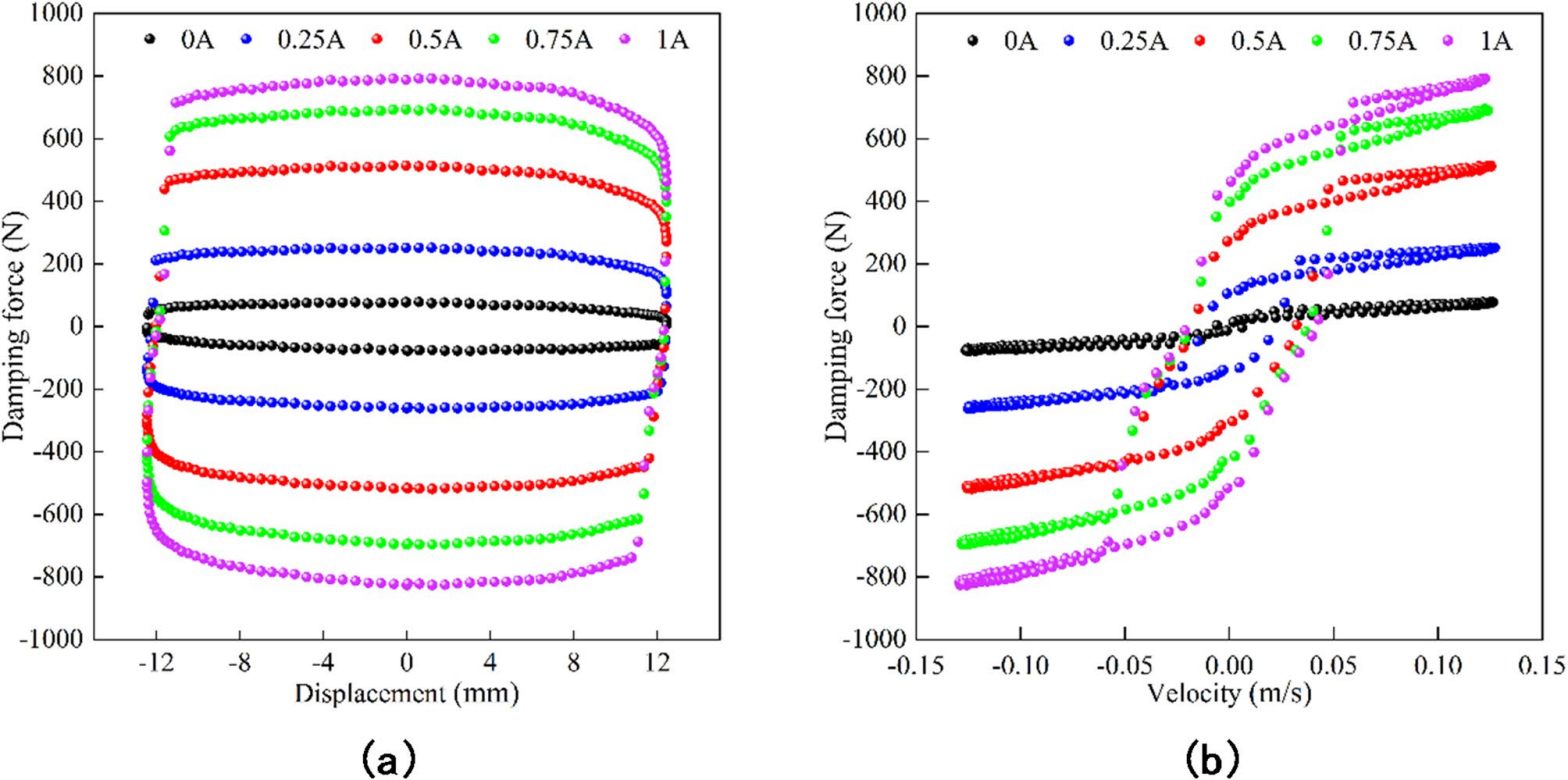
Mechanical characteristics of the MR damper under different current. (**a**) Damping force-displacement. (**b**) Damping force-velocity.

The test object is a single piston rod shear valve type MR damper designed by the research group. It combines the advantages of valve type and shear type, and has the characteristics of large output damping force, wide damping force adjustment range and large stroke. Figure 1 is the mechanical performance test system of the MR damper. A sinusoidal excitation with a frequency of 1 Hz and amplitudes of 6 mm, 8 mm, 10 mm and 12 mm was used in the test. The input current of the DC regulated power supply to the MR damper is 0 A, 0.25 A, 0.50 A, 0.75 A and 1.00 A respectively. The values of displacement$\:\:x$, velocity$\:\:v$ and damping force$\:\:F\:$ under different currents can be obtained by calculation. The indicator characteristic curve *F*-*x* and velocity characteristic curve *F*-*v* of the experimental group with amplitude of 12 mm and frequency of 1 Hz are shown in Fig. 2.

It can be seen from the curve in Fig. 2(a) that under the same excitation, the output damping force of the MR damper increases with the increase of the input current, while in the process of piston movement, the damping force does not change significantly with the displacement, and the whole is elliptical. As shown in Fig. 2(b), at the same piston velocity, the output damping force of the MR damper increases with the increase of the input current, and the curve has obvious segmentation in the low velocity and high velocity regions, which means that the velocity characteristics have some degree of nonlinear and hysteresis characteristics, especially at the low velocity stage, showing a large hysteresis loop.

### Forward modeling of the MR damper

In practical application, in order to achieve reliable control, it is necessary to establish the mathematical model of MR damper accurately and replace the damper in the simulation analysis process. The improved hyperbolic tangent model can effectively represent the hysteretic characteristics of the MR damper, avoiding the problem of parameter redundancy, and is often used in control research. In order to make the fitting results more accurately describe the dynamic characteristics of the MR damper, the modified hyperbolic tangent model is optimized by adding bias damping force, and the improved model expression is as follows:

1 $$\:F={a}_{1}{tan}h({a}_{2}(\dot{x}+kx))+{a}_{3}(\dot{x}+kx)+{f}_{0}$$

where, $\:{a}_{1}$ is the proportional factor of the hysteresis loop, which changes with the current of the coil, $\:0<{a}_{1min}<{a}_{1}<{a}_{1max}$; $\:{a}_{2}$ is the damping coefficient of the pre-yield area; $\:{a}_{3}$ is the damping coefficient of the post-yield area; $\:k$ is the proportional factor of the width of the hysteresis loop; $\:{f}_{0}$ is the bias damping force. The parameters to be identified by the algorithm in the model are:

2 $$\:\varTheta\:=\left[{a}_{1},{a}_{2},k,{a}_{3},{f}_{0}\right]$$

The mechanical properties under sinusoidal excitation with frequency of 1 Hz and an amplitude of 12 mm are selected as the identification data. The nonlinear least squares method is used to identify the parameters of the test data under the input current of 0 A, 0.25 A, 0.50 A, 0.75 A and 1.00 A of the MR damper. The principle of fitting is to find the best function matching of the best data by minimizing the square sum of the errors, it means that the sum of squares of errors between the predicted data and the actual data is minimized, so that the unknown parameter data can be easily obtained.

3 $$\:{min}e={{\sum\:}_{i=1}^{n}\left[{f}_{\text{sim}}-{f}_{\text{exp}}\right]}^{2}$$

where, $\:{f}_{\text{sim}}$ is the simulated damping force at point $\:i$; $\:{f}_{e\text{xp}}$ is the experimental damping force at point $\:i$; $\:\text{n}$ is the number of experimental points. The identification results of different current are shown in Table 1.

**Table 1:** Model parameter identification results.

Current I(A)	Parameter values				
	$\:{a}_{1}$	$\:{a}_{2}$	$\:k$	$\:{a}_{3}$	$\:{f}_{0}$
0	31.3053	59.9640	0.5001	350.4991	-2.9896
0.25	183.5885	54.4150	0.7222	535.4814	-9.6055
0.5	430.6632	32.8120	1.1443	566.5333	-10.0816
0.75	605.1327	27.8850	1.3198	598.7327	-5.9006
1.0	730.6321	25.0824	1.4341	471.7528	-4.6824

**Fig. 3:**
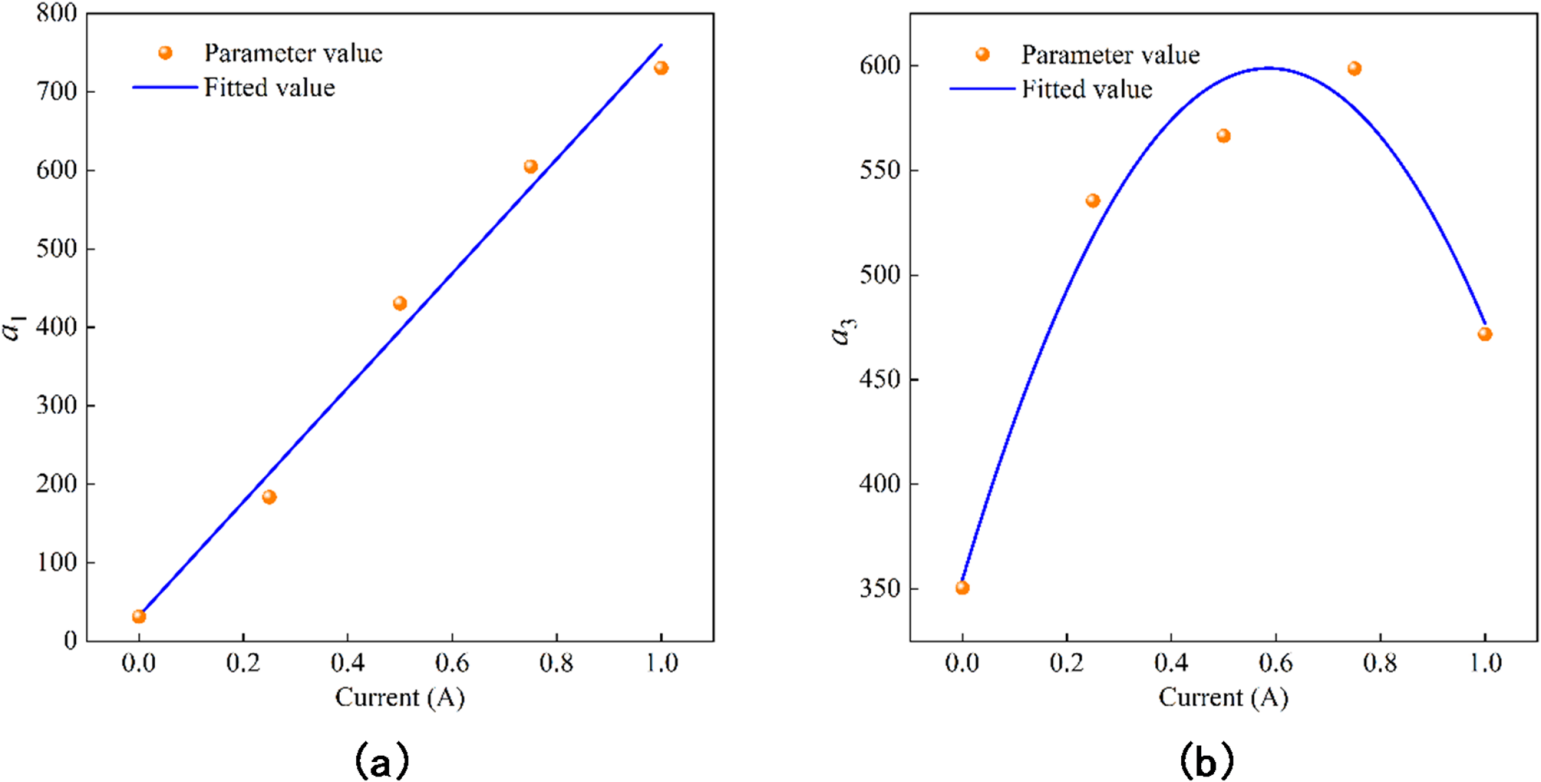
The relationship between $\:{a}_{1}$, $\:{a}_{3}$ and current. (**a**) Parameter $\:{a}_{1}$.(**b**) Parameter $\:{a}_{3}$.

By analyzing the data in the Table 1, it can be seen that the parameters $\:{a}_{2}$, $\:k$, $\:{f}_{0}$ do not change significantly under different currents, so they can be regarded as constants, and the average values $\:{a}_{2}=40.03$, $\:k=1.02$, $\:{f}_{0}=6.65$ are taken. However, the values of $\:{a}_{1}$ and $\:{a}_{3}$ will change with the increase of current. By fitting the results of parameter identification under different currents, the variation law is shown in Fig. 3.

After fitting, the expression of the relationship between $\:{a}_{1}$, $\:{a}_{3}$ and current can be obtained as follows:

4 $$\:\left\{\begin{array}{c}{a}_{1}={b}_{1}I+{c}_{1}=728.08I+32.22\\\:{a}_{3}={b}_{2}{I}^{2}+{c}_{2}I+d=-711.75{I}^{2}+834.05I+354.48\end{array}\right.$$

At this point, the five parameters of the improved hyperbolic tangent model can be determined, and the expression of the improved hyperbolic tangent model of the identified MR damper is

5 $$\:F=({b}_{1}I+{c}_{1}){tan}h({a}_{2}(\dot{x}+kx))+({b}_{2}{I}^{2}+{c}_{2}I+d\left)\right(\dot{x}+kx)+{f}_{0}$$

**Fig. 4:**
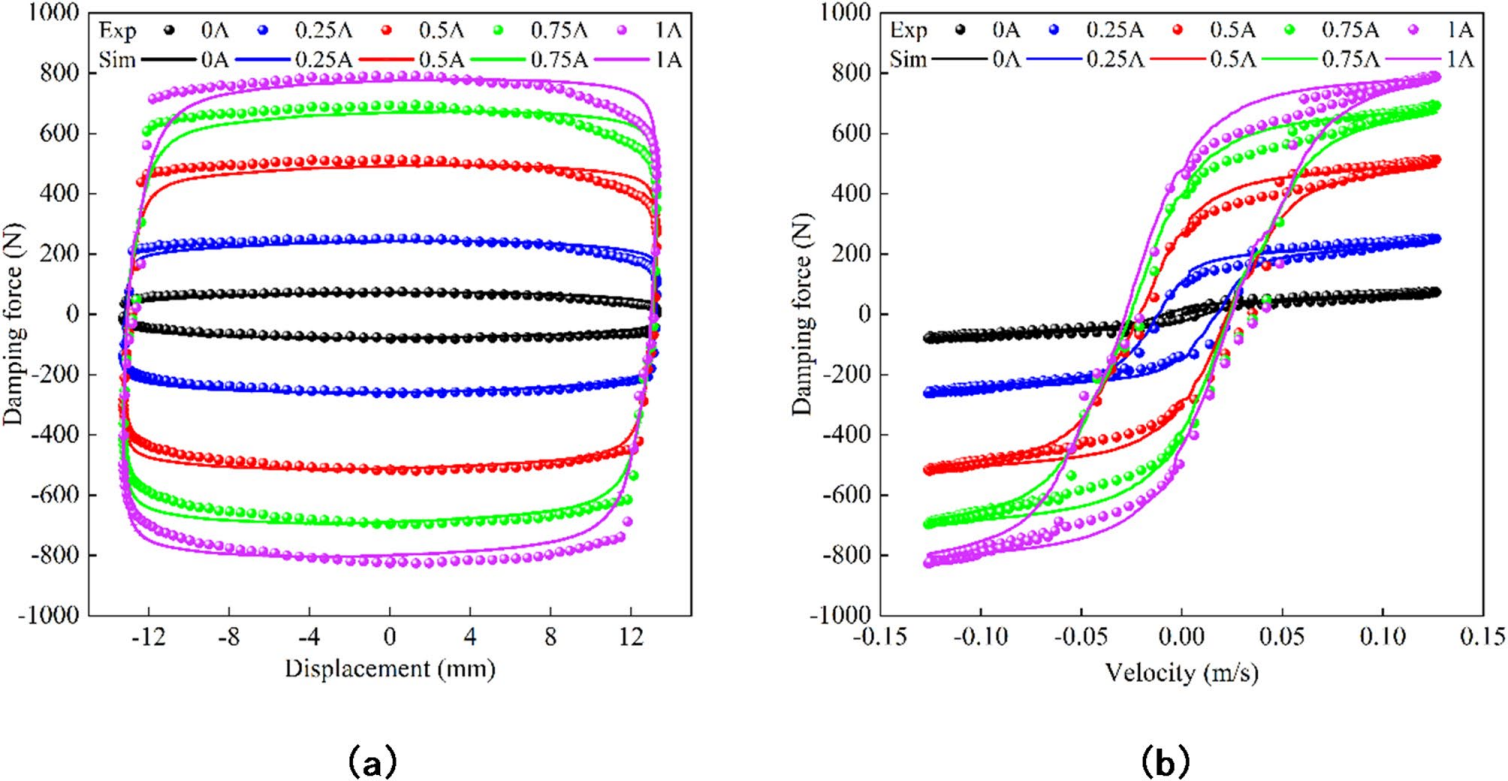
The simulation results are compared with the experimental results. (**a**) Damping force-displacement. (**b**) Damping force-velocity.

In this paper, sinusoidal excitation with amplitude of 12 mm and frequency of 1 Hz is used to simulate and analyze the improved hyperbolic tangent model Eq. (5). The simulation results show the characteristic curves of the MR damper under different current conditions, and are compared with the mechanical response curves obtained by the test as shown in Fig. 4. The results show that the characteristic curves of the improved hyperbolic tangent model after parameter identification are basically consistent with the experimental curves of the MR damper, which can effectively reflect the nonlinear hysteresis characteristics of the MR damper under low velocity and high velocity. Compared with other complex models, this model not only meets the more accurate simulation requirements, but also has a simple structure and fewer parameters to be identified. It has a good application prospect, especially in the semi-active suspension control system.

### Inverse modeling of the MR damper

**Fig. 5:**
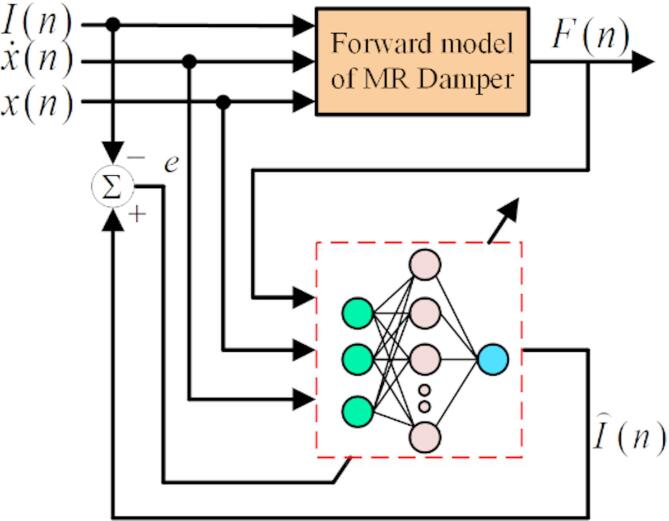
CNN inverse model of MR damper.

Convolutional Neural Networks (CNN) is a multi-layer feedforward neural networks containing convolution calculation^32^. In this paper, CNN is used to establish the inverse model of the MR damper. The principle is shown in Fig. 5. The experimental data of displacement, velocity and damping force under specific current of the damper are used as the input of CNN, and the control current of the MR damper is inferred inversely. The system corrects the model continuously according to the error between the inference current and the test current of the inverse model until the model reaches a certain accuracy.

**Fig. 6:**
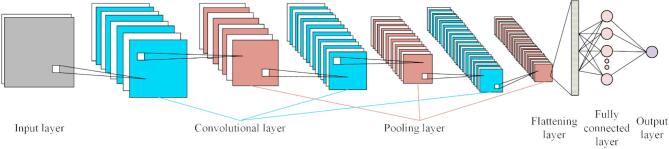
Convolutional neural network structure.

In order to improve the accuracy of the model, 3365 sets of test data of the MR damper within 0–2 A are divided into training set and test set, the ratio is 7:3, and 20% of the data set is taken as verification set. The training cycle of the training set is divided into 500 rounds, each round carries out 18 iterations, a total of 9000 iterations, and the initial learning rate is 0.001. According to the simulation of sample data, a total of 10 layers of network model are set up, including 1 input layer, 3 convolutional layers, 3 pooling layers, 1 flattening layer, 1 fully connected layer and 1 output layer respectively. The structure is shown in Fig. 6.

**Fig. 7:**
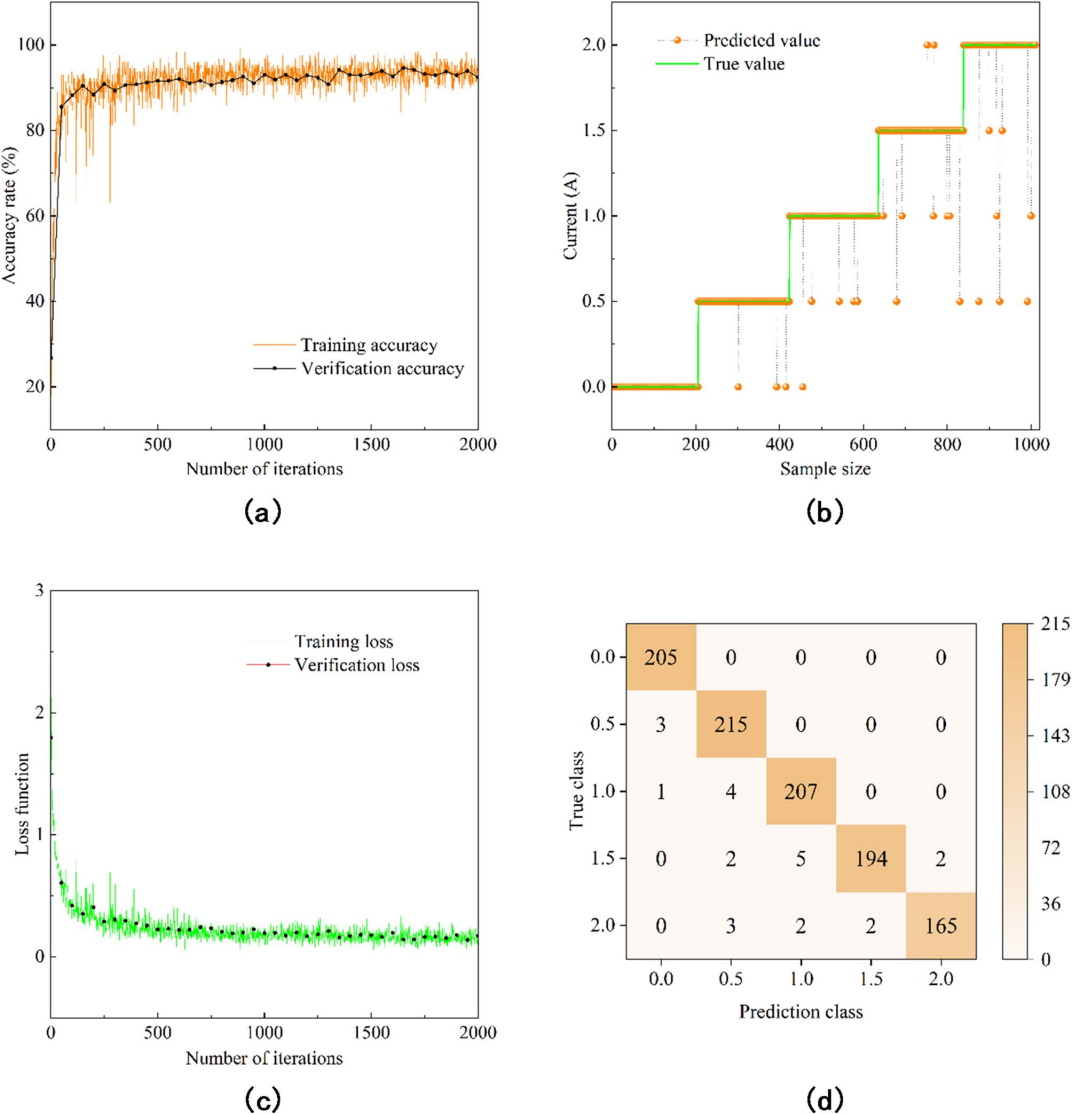
CNN Training, testing and verification. (**a**) Accuracy rate. (**b**) Comparison of test set prediction results. (**c**) Loss function. (**d**) Confusion Matrix for Test Data.

Figure 7(a) and (c) show the accuracy and loss curves of part of the training set and verification set, in which the accuracy of the training set and verification set is close to 100%, the loss value is close to 0, and no overfitting phenomenon occurs in the training. Table 6 shows the accuracy of each data set under CNN training, in which the training set is 97.83%, the verification set is 97.88%, and the test set is 97.62%. Figure 7(b) is the comparison between the predicted value and the true value of the test set, and Fig. 7(d) is the confusion matrix of the test set results, which can be seen that 24 sample identification errors occurred in the process of state identification of the test sample, but the accuracy rate of the test set in Table 2 is 97.62%. Therefore, CNN has a good recognition effect.

**Table 2:** Accuracy of different data sets.

Data set	Accuracy rate
Training set	97.83%
Validation set	97.88%
Test set	97.62%

In addition to the accuracy rate, the precision rate $\:P$, recall rate $\:R$ and $\:{F}_{1}$ value can also be used to evaluate the performance of CNN, which is defined as:

6 $$\:{P}_{j}=\frac{T{P}_{j}}{T{P}_{j}+F{P}_{j}},(j=\text{1,2},\text{3,4},5)$$

7 $$\:{R}_{j}=\frac{T{P}_{j}}{T{P}_{j}+F{N}_{j}}$$

8 $$\:{{F}_{1}}_{j}=\frac{2{P}_{j}{R}_{j}}{{P}_{j}+{R}_{j}}$$

where, True Positive (TP) is the true positive example, the actual value is true, and the predicted value is also the true number of samples; False Negative (FN) is the false negative example, the actual is true, and the predicted number of samples is negative; False Positive (FP) is the false positive example, the actual is false, and the predicted number of samples is true; True Negative (TN) is the true negative example, the actual is true, and the predicted number of samples is false. The basic structure of the confusion matrix is shown in Table 3.

**Table 3:** Confusion matrix basic structure.

	Prediction is positive	Prediction is negative
Truth is positive	TP	FN
Truth is negative	FP	TN

**Fig. 8:**
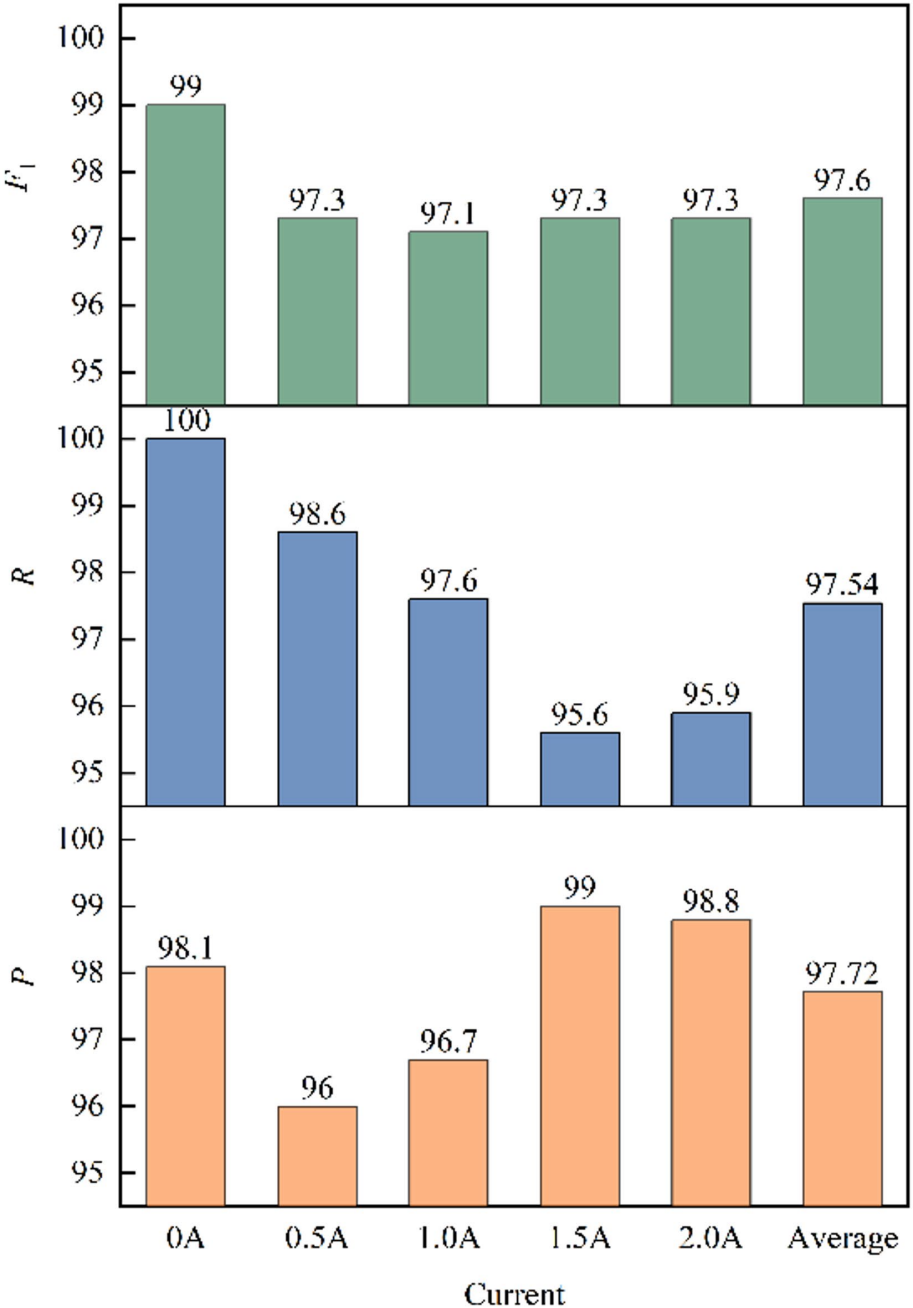
Test result evaluation index.

The precision rate $\:P$, recall rate $\:R$ and $\:{F}_{1}$ value under each current are as shown in Fig. 8. The precision rate and recall rate under the same current are high and close, and the $\:{F}_{1}$ value is correspondingly high. It has high precision rate and recall rate under different currents, indicating that the model has a consistent and good effect on different currents. The average $\:{F}_{1}$ value is 97.6%, which proves that the network can achieve accurate classification of different currents and has good reasoning accuracy. Therefore, the established CNN inverse model of MR damper can be used for the control research of magnetorheological semi-active suspension system.

### Modeling of quarter MR semi-active suspension with air spring

#### Air spring model

**Fig. 9:**
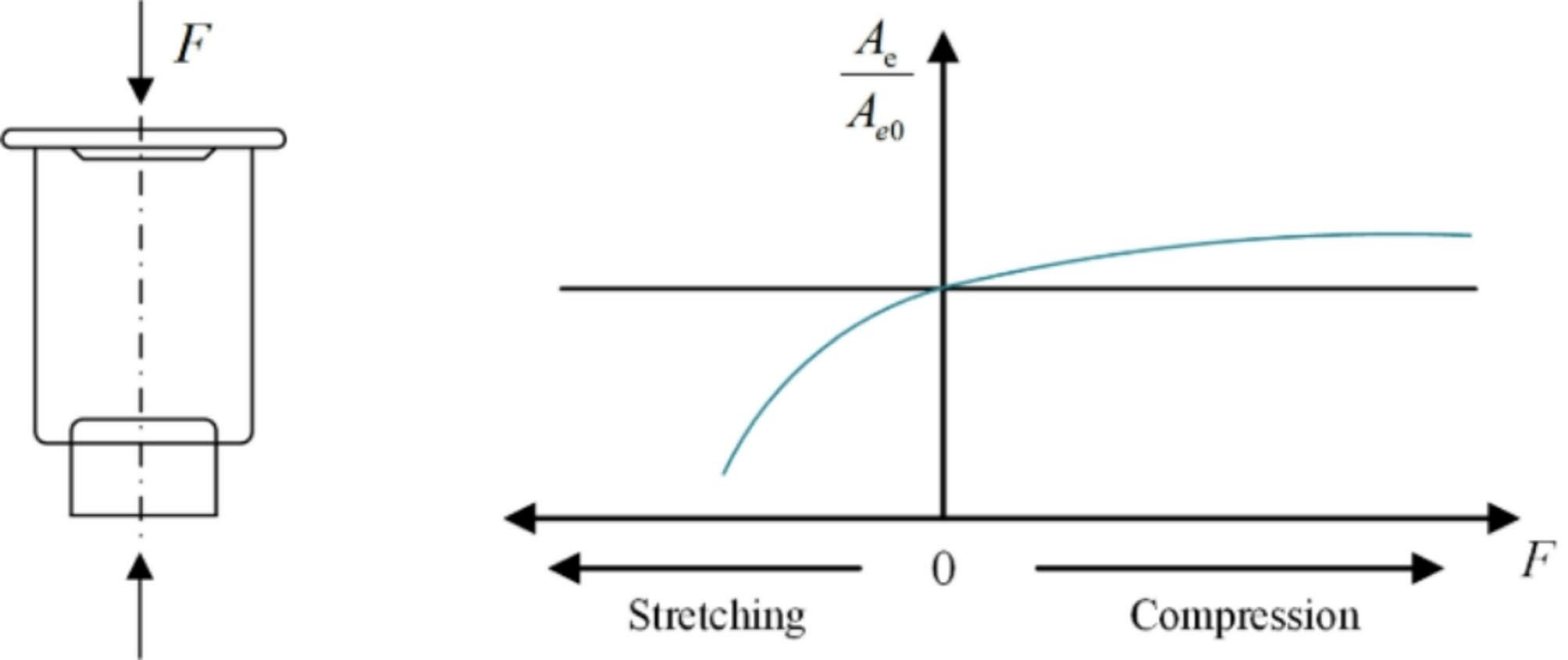
Structure and characteristic curve of membrane air spring.

When the load of the vehicle changes, the air spring can change the inflation pressure and adjust the height of the vehicle to make the vibration frequency of the suspension unchanged. This is the aspect that the air spring is obviously superior to the metal spring. The membrane air spring can obtain a lower spring stiffness without an auxiliary chamber, and the effective area change rate can be controlled by changing the shape and size of the piston to obtain an ideal inverse S curve. Figure 9 shows the structure and characteristic curve of the membrane air spring. During compression, the effective area change rate of the airbag gradually decreases.

For the driving vehicle, the road excitation cannot be changed at will, but the comfort can be improved by the control of the suspension system. The air spring uses the reaction force of compressed air in the rubber air bag as the elastic restoring force, and the vertical load generated by the air spring is:

9 $$\:F=P{A}_{e}$$

where, $\:P\:$is the pressure in the air bag; $\:{A}_{e}\:$is the effective area.

The air spring carries out heat exchange with the outside world by vertically stretching or compressing the internal gas. However, due to the vibration excitation of the air suspension, it is assumed that there is no heat exchange between the internal gas of the air spring and the outside world, that is, the thermodynamic transformation of the internal gas of the air spring is an adiabatic process, and the gas equation of state at this time is:

10 $$\:(P+{P}_{a}){{V}_{e}}^{n}=({P}_{0}+{P}_{a}){{V}_{0}}^{n}$$

where, $\:{V}_{e}$ is the working volume; $\:{V}_{0}$ is the initial volume; $\:{P}_{a}$ is the standard atmospheric pressure and $\:n$ is the thermodynamic index.

According to the Boyle ‘s law under adiabatic conditions, the pressure $\:P$ in the air spring airbag can be obtained as:

11 $$\:P=({P}_{0}+{P}_{a})({V}_{0}/{V}_{e}{)}^{n}-{P}_{a}$$

The combinatorics Eq. (9) and Eq. (11) knowable Eq. (12) are as follows:

12 $$\:F=\left(\right({P}_{0}+{P}_{a}\left)\right({V}_{0}/{V}_{e}{)}^{n}-P){A}_{e}$$

Due to the nonlinear stiffness characteristic of air spring in the process of vehicle driving. In theoretical calculations, the stiffness of air spring can be obtained directly by derivation of vertical load $\:F$ to displacement $S$, take the derivative of the Eq. (12):

13 $$\:{k}_{s}=\frac{dF}{ds}=P\frac{d{A}_{e}}{ds}-{A}_{e}\left(\frac{n({P}_{0}+{P}_{a}){{V}_{0}}^{n}}{{V}_{e}^{n+1}}\right)\frac{d{V}_{e}}{ds}$$

When in equilibrium, there is:

14 $$\:P={P}_{0}, {V}_{e}={V}_{0}$$

Then, the stiffness of the air spring in the equilibrium state is:

15 $$\:{k}_{0}={P}_{0}\frac{d{A}_{e}}{ds}-{A}_{e}\frac{n}{{V}_{0}}({P}_{0}+{P}_{a})\frac{d{V}_{0}}{ds}$$

#### Quarter suspension dynamics model

**Fig. 10:**
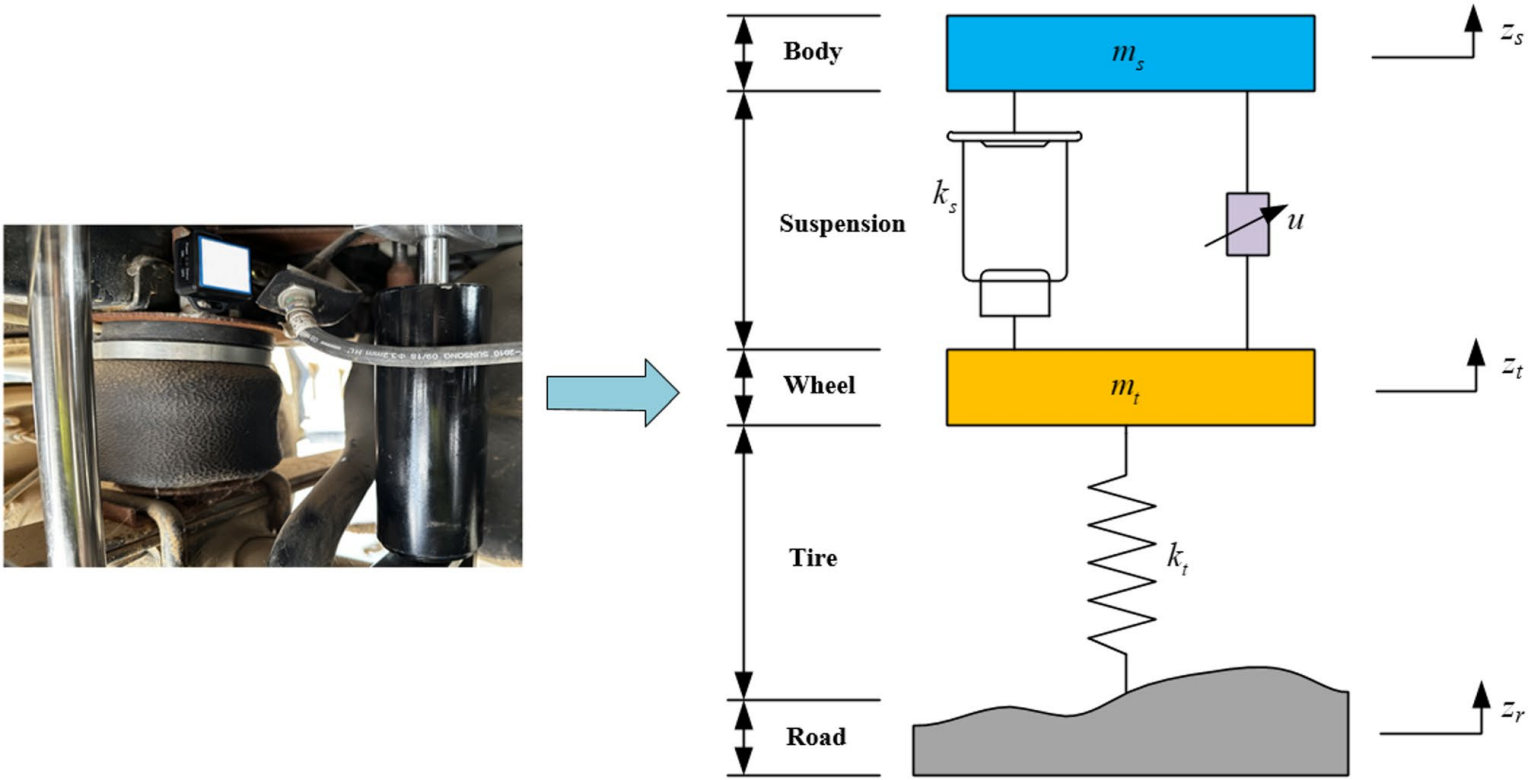
Quarter MR semi-active suspension with air spring model.

This paper focuses on the two-degree-of-freedom quarter MR semi-active suspension with air spring. The simplified model is shown in Fig. 10, which is mainly composed of body, tire, air spring, MR damper and other components. Compared with other models, this model has a simple structure and is easy to collect signals. It can better show the vibration effects of suspension vertical motion and road input on handling stability and ride comfort.

Establishing differential equations:

16 $$\:\left\{\begin{array}{c}{m}_{s}{\ddot{z}}_{s}+{k}_{s}({z}_{s}-{z}_{t})-u=0\\\:{m}_{t}{\ddot{z}}_{t}+{k}_{s}({z}_{t}-{z}_{s})+{k}_{t}({z}_{t}-{z}_{r})+u=0\end{array}\right.$$

where, $\:{m}_{s}\:$is the body mass; $\:{m}_{t}$ is the wheel mass; $\:{k}_{s}\:$is the air spring stiffness; $\:{k}_{t}$ is the tire stiffness; $\:u\:$is the output damping force of the MR damper; $\:{z}_{r}$ is the displacement of the road excitation; $\:{z}_{s}$ is the vertical displacement of the body and $\:{z}_{t}$ is the vertical displacement of the wheel.

According to Eq. (16), the state vector of the system is selected as:

17 $$\:Z={\left[\begin{array}{cccc}{z}_{s}&\:{\dot{z}}_{s}&\:{z}_{t}&\:{\dot{z}}_{t}\end{array}\right]}^{T}$$

The output vector is:

18 $$\:Y={\left[\begin{array}{ccc}{\ddot{z}}_{s}&\:{z}_{s}-{z}_{t}&\:{z}_{t}-{z}_{r}\end{array}\right]}^{T}$$

The input vector consists of the damper output damping force $\:u\:$ and the pavement excitation displacement $\:{z}_{r}$:

19 $$\:U={\left[\begin{array}{cc}u&\:{z}_{r}\end{array}\right]}^{T}$$

Then the state space expression is:

20 $$\:\left\{\begin{array}{c}\dot{Z}=AZ+BU\\\:Y=CZ+DU\end{array}\right.$$

where,

$$\:A=\left[\begin{array}{cccc}0&\:1&\:0&\:0\\\:-\frac{{k}_{s}}{{m}_{s}}&\:0&\:\frac{{k}_{s}}{{m}_{s}}&\:0\\\:0&\:0&\:0&\:1\\\:\frac{{k}_{s}}{{m}_{t}}&\:0&\:-\frac{{k}_{s}+{k}_{t}}{{m}_{t}}&\:0\end{array}\right],\:B=\left[\begin{array}{cc}0&\:0\\\:\frac{1}{{m}_{s}}&\:0\\\:0&\:0\\\:-\frac{1}{{m}_{t}}&\:\frac{{k}_{t}}{{m}_{t}}\end{array}\right],\:C=\left[\begin{array}{cccc}-\frac{{k}_{s}}{{m}_{s}}&\:0&\:\frac{{k}_{s}}{{m}_{s}}&\:0\\\:1&\:0&\:-1&\:0\\\:0&\:0&\:1&\:0\end{array}\right],\:D=\left[\begin{array}{cc}\frac{1}{{m}_{s}}&\:0\\\:0&\:0\\\:0&\:-1\end{array}\right].$$

### Controller design

**Fig. 11:**
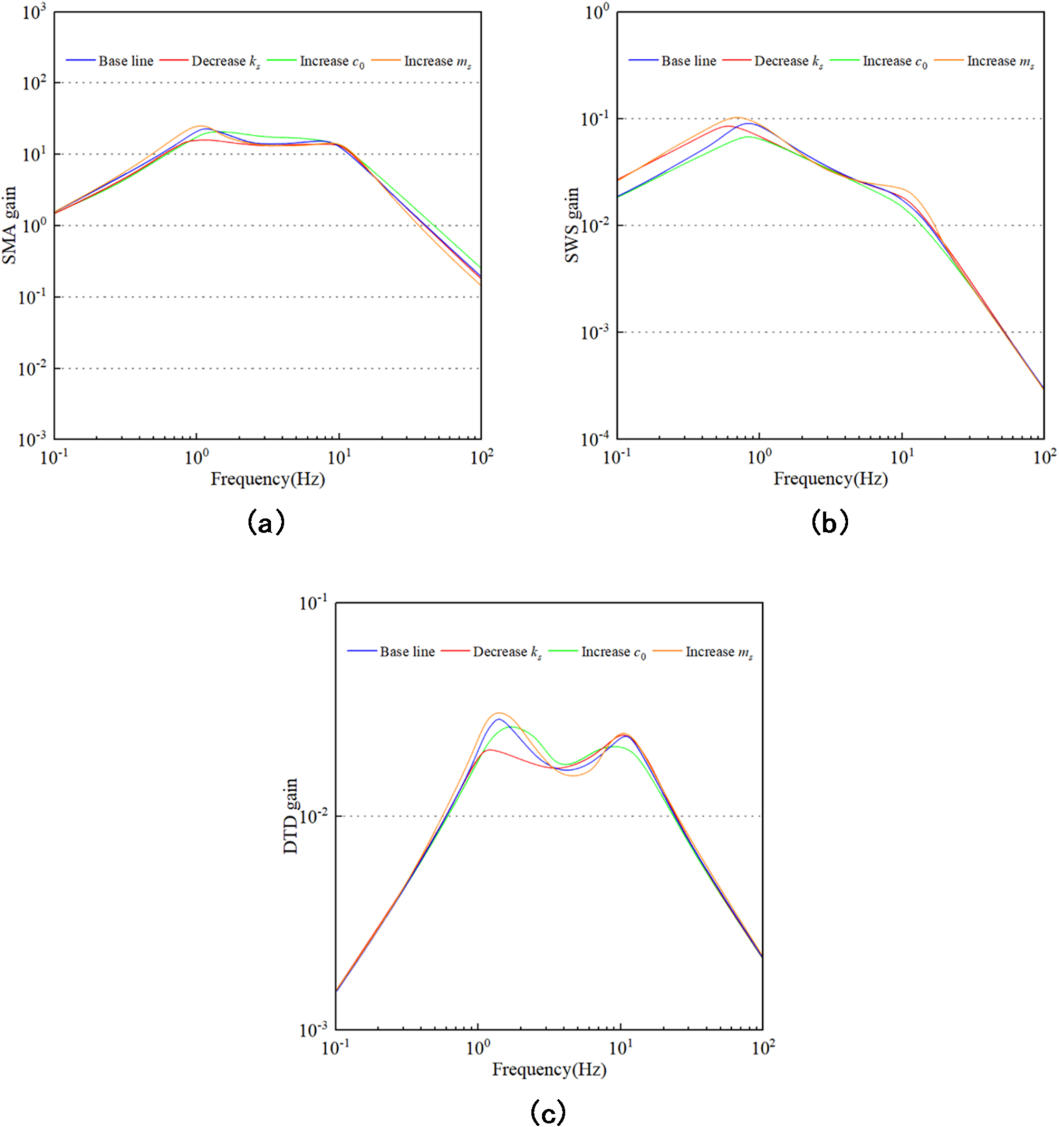
Sensitivity analysis.

To analyze the sensitivity impact of the uncertainty of suspension parameter uncertainty on system performance, the influence of the changes in relevant parameters on the performance of the automotive suspension can be reflected by the Bode diagram. Figure 11(a), (b), and (c) respectively reflect the frequency response of the automotive suspension when the suspension stiffness $\:{k}_{s}$ is reduced, the zero-field damping $\:{c}_{0}$ is increased, and the spring mass $\:{m}_{s}$ is increased. As can be seen from Fig. 11, the sprung mass $\:{m}_{s}$ and the zero-field damping $\:{c}_{0}$ of the damper have a significant impact on the human body’s sensitive frequency range of vehicle body acceleration. However, for the dynamic travel of the suspension, only in the low-frequency range are the sensitivity of the sprung mass $\:{m}_{s}$ and the spring stiffness $\:{k}_{s}$ of the suspension relatively strong. For the dynamic travel of the tire, the sensitivity of the sprung mass $\:{m}_{s}$ and the zero-field damping $\:{c}_{0}$ of the damper is relatively strong. In conclusion, from the perspective of ride comfort, the sprung mass $\:{m}_{s}$ and the suspension spring stiffness $\:{k}_{s}$ are important influencing parameters for ride comfort. From the perspective of handling safety, the influence of tire stiffness $\:{k}_{t}$ cannot be ignored. Therefore, this paper selects the sprung mass $\:{m}_{s}$ as the variation parameter for the subsequent controller design, thereby improving vehicle performance.

**Fig. 12:**
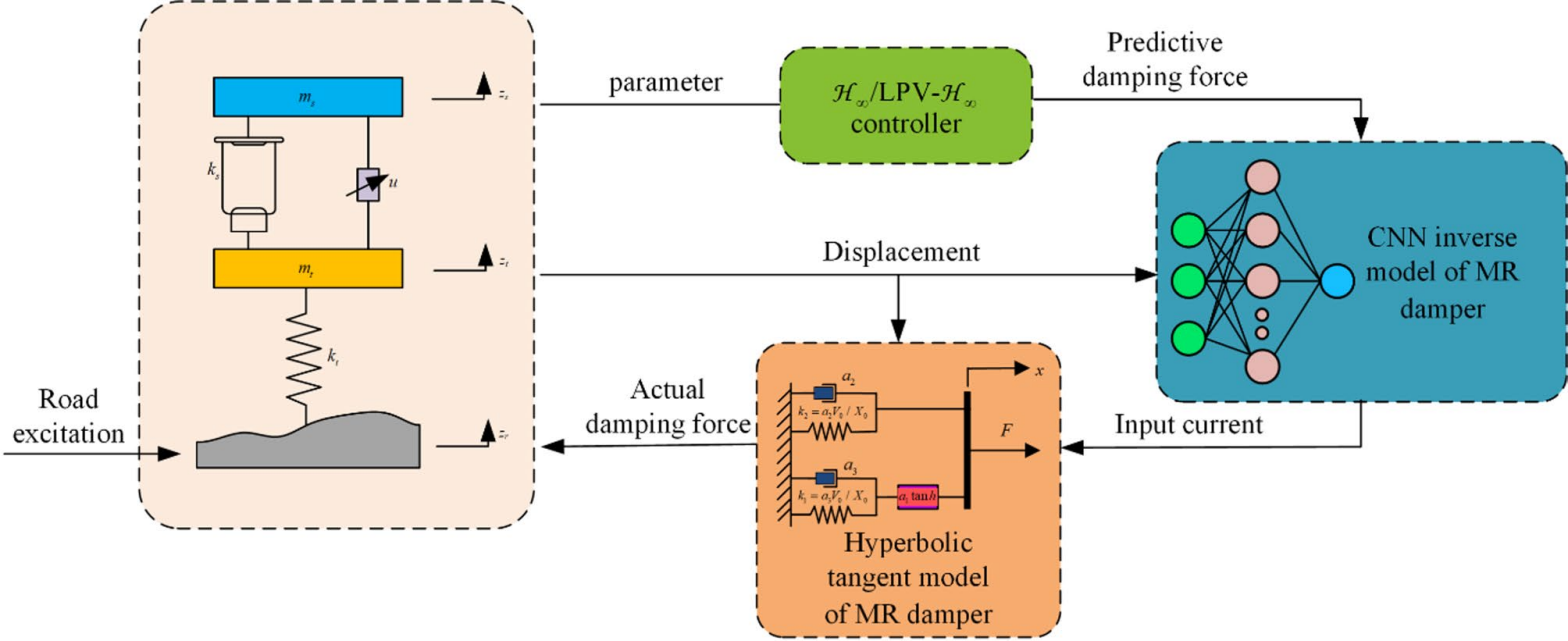
The control principle diagram of the suspension system.

The control principle diagram of the MR semi-active suspension with air spring is shown in Fig. 12. The controller analyzes the dynamic behavior of the suspension according to the parameters of the suspension under road excitation to predict the required damping force. The control current is obtained through the CNN inverse model and input to the MR damper to generate the actual damping force.

When designing the controller, it is necessary to make the following analysis of the control objectives:

- The weighted acceleration root mean square value is used to characterize the ride comfort of the vehicle, and the smaller the value, the better.
- The relative dynamic load of the wheel is used to characterize the safety of the vehicle, and the value is required to be less than 1.
- The suspension travel is less than the maximum travel of the actuator or the specified amplitude to avoid hitting the limit block.
- The vibration control force should be less than the maximum control force output by the actuator.

Based on the above control objectives, the design of suspension vibration control optimization algorithm is constrained:

- The vehicle body acceleration is reduced by reducing the H_∞_ norm of the transfer function $\:G_{{\ddot{z}_{1} \sim {\text{ q}}}}$ of the road disturbance to the vehicle body acceleration.
- The wheel dynamic load is less than the static load.
- The suspension dynamic stroke is less than the maximum stroke of the actuator.
- The vibration control force is less than its maximum value.

The specific performance is:

21 $$\:\left\{ {\begin{array}{*{20}c} {sup\left\| {G_{{\ddot{z}_{s} \sim z_{r} }} } \right\|_{{\infty \:}} } & { < \gamma \:_{{min}} } \\ {\:\left| {\frac{{k_{t} (z_{t} - z_{r} )}}{{(m_{s} + m_{t} )}}} \right|} & { < 1} \\ {\:\left| {\frac{{z_{s} - z_{t} }}{{z_{{max}} }}} \right|} & { < 1} \\ {\:\left| {\frac{{u\left( t \right)}}{{u_{{max}} }}} \right|} & { < 1} \\ \end{array} } \right.$$

According to the above control objectives and constraints, the system state variable $\:x\left(t\right)$, the system control output $\:{z}_{\infty\:}\left(t\right)$, and the system observation output $\:y\left(t\right)$ are respectively:

22 $$\:\left\{\begin{array}{c}x\left(t\right)={\left[\begin{array}{cccc}{z}_{s}-{z}_{t}&\:{\dot{z}}_{s}&\:{z}_{t}-{z}_{r}&\:{\dot{z}}_{t}\end{array}\right]}^{T}\\\:{z}_{\infty\:}\left(t\right)={\ddot{z}}_{s}\\\:y\left(t\right)={\left[\begin{array}{ccc}\frac{{z}_{s}-{z}_{t}}{{z}_{max}}&\:\frac{{k}_{t}({z}_{t}-{z}_{r})}{({m}_{s}+{m}_{t})g}&\:\frac{u}{{u}_{max}}\end{array}\right]}^{T}\end{array}\right.$$

It is expressed in state space form:

23 $$\:\left\{\begin{array}{c}\dot{x}\left(t\right)={A}_{1}x\left(t\right)+{B}_{1}u\left(t\right)+{B}_{2}\left(t\right)w\left(t\right)\\\:{z}_{\infty\:}\left(t\right)={C}_{1}x\left(t\right)+{D}_{1}u\left(t\right)\\\:y\left(t\right)={C}_{2}x\left(t\right)+{D}_{2}u\left(t\right)\end{array}\right.$$

where,

$$\:{A}_{1}=\left[\begin{array}{cccc}0&\:1&\:0&\:-1\\\:-\frac{{k}_{s}}{{m}_{s}}&\:0&\:0&\:0\\\:0&\:0&\:0&\:1\\\:\frac{{k}_{s}}{{m}_{t}}&\:0&\:-\frac{{k}_{t}}{{m}_{t}}&\:0\end{array}\right],\:{B}_{1}=\left[\begin{array}{c}0\\\:\frac{1}{{m}_{s}}\\\:0\\\:-\frac{1}{{m}_{t}}\end{array}\right],{\:B}_{2}=\left[\begin{array}{c}0\\\:0\\\:-1\\\:0\end{array}\right],\:{C}_{1}=\left[\begin{array}{cccc}-\frac{{k}_{s}}{{m}_{s}}&\:0&\:0&\:0\end{array}\right],\:{D}_{1}=\left[\frac{1}{{m}_{s}}\right],$$

$$\:{C}_{2}=\left[\begin{array}{cccc}\frac{1}{{z}_{max}}&\:0&\:0&\:0\\\:0&\:0&\:\frac{{k}_{t}}{({m}_{s}+{m}_{t})g}&\:0\\\:0&\:0&\:0&\:0\end{array}\right],\:{D}_{2}=\left[\begin{array}{c}0\\\:0\\\:\frac{1}{{u}_{max}}\end{array}\right],\:w\left(t\right)=\left[{\dot{z}}_{r}\right].$$

### Design of H_∞_ controller

**Fig. 13:**
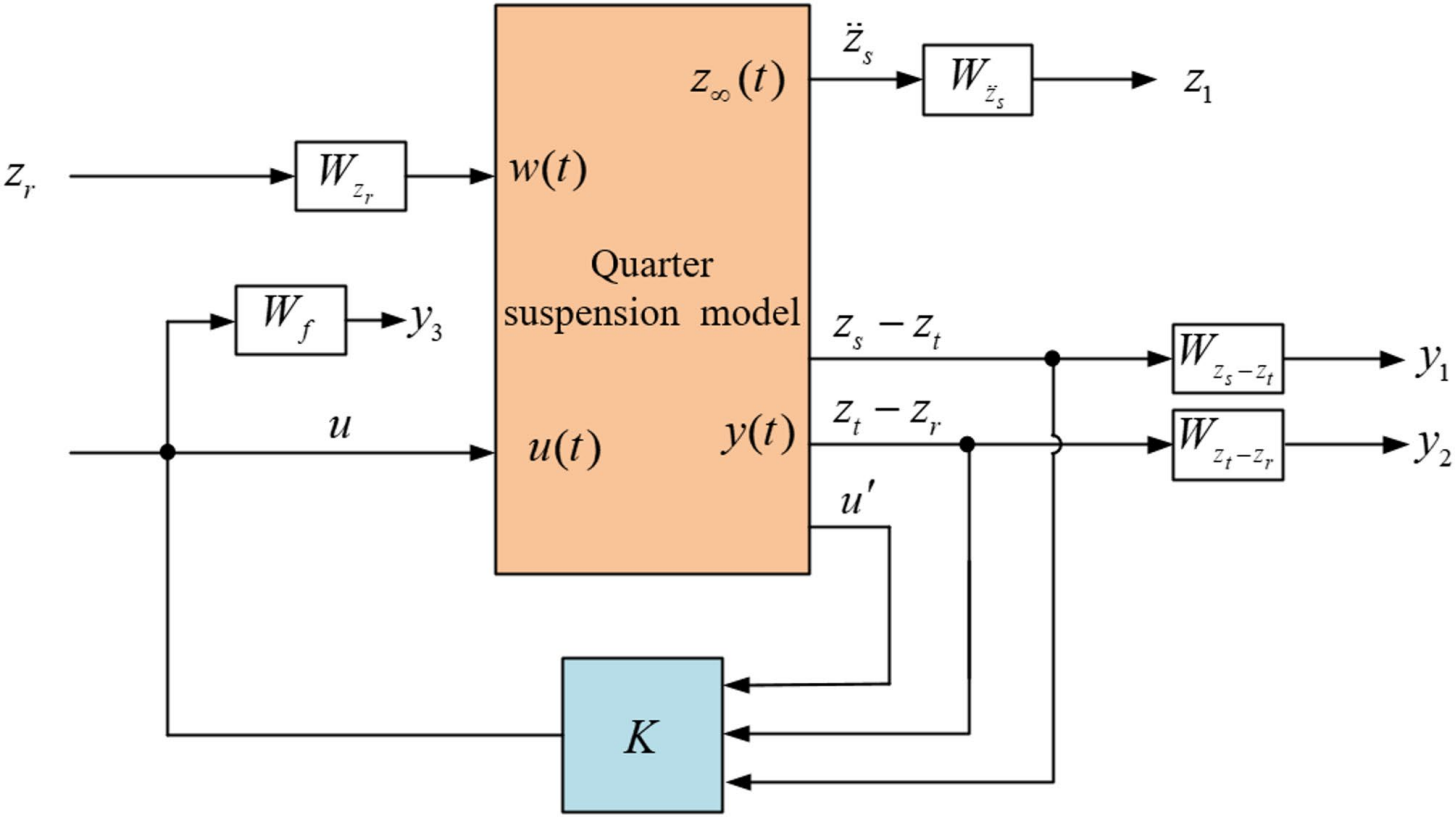
H_∞_ controller structure.

In the H_∞_ controller structure Fig. 13, the disturbance *w* will obviously affect the output $\:{z}_{{\infty\:}}$ and *y*, and it is impossible to completely eliminate the influence of *w* on *x*, $\:{z}_{{\infty\:}}\:$and *y*. Therefore, it is necessary to design a state feedback controller $\:u\left(t\right)=Kx\left(t\right)$ to make the system stable.

To achieve the optimal suspension performance, it is necessary to perform weighting processing on the system. According to the ISO2631 standard, the frequency range that the human body is most sensitive to is 4 to 8 Hz. Therefore, the vehicle body acceleration is frequency-weighted. Based on the “fatigue-reduced efficiency limit” curve given by the standard for the human body to withstand vertical vibration, if it is desired for the vehicle body acceleration frequency to be close to the “fatigue-reduced efficiency limit” curve, Then the reciprocal amplitude-frequency characteristic of the vehicle body acceleration weighting function should be similar to the “fatigue-reduced efficiency limit” curve.

So the weighting function of the Sprung mass acceleration(SMA) is as follows

$$\:{W}_{z}=\frac{s+20}{{s}^{2}+55s+2285}$$

To ensure that the dynamic travel of the suspension does not exceed the maximum working space of the suspension and that the dynamic displacement of the tires is limited within the maximum deformation range of the tires. The weighting functions of Suspension working space (SWS) and Dynamic tire deformation (DTD) are

$$\:{W}_{{z}_{s}-{z}_{t}}={W}_{{z}_{t}-{z}_{r}}=\frac{25}{0.2s+1}$$

The output force weighting function of the MR damper is

$$\:{W}_{u}=0.001$$

The weighting function of road surface excitation is

$$\:{W}_{{z}_{r}}=0.07$$.

Then the closed-loop system is:

24 $$\:\left\{\begin{array}{c}\dot{x}\left(t\right)=\overline{A}x\left(t\right)+\overline{B}w\left(t\right)\\\:{z}_{\infty\:}\left(t\right)=\overline{{C}_{1}}x\left(t\right)\\\:y\left(t\right)=\overline{{C}_{2}}x\left(t\right)\end{array}\right.$$

where,$\:\overline{A}={A}_{1}+{B}_{1}K$,$\:\:\overline{B}={B}_{2}$, $\:\overline{{C}_{1}}={C}_{1}+{D}_{1}K$, $\:\overline{{C}_{2}}={C}_{2}+{D}_{2}K$; $\:{z}_{\infty\:}$is the evaluation output under H_∞_ performance index.

Define the closed-loop transfer function of *w* on $\:{z}_{{\infty\:}}$ as $\:{T}_{zw}=\frac{{z}_{{\infty\:}}}{w}$, the closed-loop transfer function of *w* on *y* as $\:{T}_{yw}=\frac{y}{w}$,Then

25 $$\:\left\| {T_{{zw}} } \right\| = \mathop {sup}\limits_{{w \ne \:0}} \frac{{\left\| {z_{{\infty \:}} } \right\|_{2} }}{{\left\| w \right\|_{2} }},\:\left\| {T_{{yw}} } \right\| = \mathop {sup}\limits_{{w \ne \:0}} \frac{{\left\| y \right\|_{2} }}{{\left\| w \right\|_{2} }}$$

Therefore, the influence of *w* on $\:{z}_{{\infty\:}}$ is limited to a certain range. For a given $\:\gamma\:>0$, design a state feedback controller $\:u\left(t\right)=Kx\left(t\right)$ so that:

26 $$\left\| {T_{{zw}} \left( {j\omega \:} \right)} \right\|_{{\infty \:}} < \gamma ,\left\| {T_{{yw}} \left( {j\omega \:} \right)} \right\|_{{\infty \:}} < \gamma \:$$

where, $\:\omega\:$ is the frequency of concern, $\:\omega\:\in\:({\omega\:}_{1},{\omega\:}_{2})$.

The Lyapunov function method is the most fundamental approach for stability analysis and controller design in control theory. If the quadratic form $\:V\left(x\right)={x}^{T}Px$ is taken as the Lyapunov function, then there is $\:\dot{V}\left(x\right)={\dot{x}}^{T}Fx+{x}^{T}F\dot{x}={x}^{T}(\:{\overline{A}}^{T}F+F\overline{A})x$. Define $\:{\overline{A}}^{T}F+F\overline{A}=-Q$, then we can obtain $\:\dot{V}\left(x\right)=-{x}^{T}Qx<0$. Therefore, for LTI systems, it is easy to apply the equation $\:{\overline{A}}^{T}F+F\overline{A}=-Q$ to obtain the Lyapunov function $\:V\left(x\right)={x}^{T}Px$, thereby determining the stability of the system.

For a given constant $\:\gamma\:$, $\:\eta\:$and $\:\rho\:$, if a closed-loop uncertain system exists a Lyapunov function $\:V\left(x\right)={x}^{T}Px$, There exist symmetric positive definite matrices $\:P>0$, $\:F>0$,$\:\:Q>0$ satisfying the condition of negative definite matrices such that:

27 $$\:{\overline{A}}^{T}F+F\overline{A}<0$$

Then the LMI set is established

28 $$\:\left[\begin{array}{ccc}{\overline{A}}^{T}F+F\overline{A}&\:F\overline{B}&\:{\overline{{C}_{1}}}^{T}\\\:{\overline{B}}^{T}F&\:-\eta\:I&\:0\\\:\overline{{C}_{1}}&\:0&\:-\eta\:I\end{array}\right]<0$$

29 $$\:\left[\begin{array}{cccc}-Q&\:P+j{\omega\:}_{c}Q-F&\:0&\:0\\\:P-j{\omega\:}_{c}Q-F&\:-{\omega\:}_{1}{\omega\:}_{2}Q+{\left[F\overline{A}\right]}_{s}&\:F\overline{B}&\:{\overline{{C}_{1}}}^{T}\\\:0&\:{\overline{B}}^{T}F&\:-{\gamma\:}^{2}I&\:0\\\:0&\:\overline{{C}_{1}}&\:0&\:-I\end{array}\right]<0$$

30 $$\:\left[\begin{array}{cc}-I&\:\sqrt{\rho\:}K\\\:*&\:-{u}_{max}^{2}F\end{array}\right]\le\:0$$

31 $$\:\left[\begin{array}{cc}-I&\:\sqrt{\rho\:}{\left\{\overline{{C}_{2}}\right\}}_{i}\\\:*&\:-F\end{array}\right]<0,i=\text{1,2}$$

where,* P* is the symmetric positive definite matrix corresponding to the performance index H_∞_, and $\:I$ is the identity matrix. $\:{\omega\:}_{c}=({\omega\:}_{1}+{\omega\:}_{2})/2$.

Considering that inequality (28) and (29) contain nonlinear terms, which cannot be solved directly by LMI toolbox in MATLAB, the following transformation is carried out. Hypothesis:

32 $$\:\left\{\begin{array}{c}{J}_{1}=\text{diag}\left\{{F}^{-1},I,I\right\}\\\:{J}_{2}=\text{diag}\left\{{F}^{-1},{F}^{-1},I,I\right\}\\\:{J}_{3}=\text{diag}\left\{I,{F}^{-1}\right\}\end{array}\right.$$

The matrix $\:{J}_{1}$, $\:{J}_{2},\:{J}_{3}$ and $\:\:{J}_{3}$ are used for congruent transformation of Eqs. (28), (29), (30) and (31) by left multiplying them and right multiplying their transposes respectively, and the variables are defined that $\:\overline{P}={F}^{-1}P{F}^{-1}$,$\:\overline{Q}={F}^{-1}Q{F}^{-1}$, $\:\overline{F}={F}^{-1}$, $\:\overline{K}=K{F}^{-1}$. Taking $\:\overline{P}$, $\:\overline{Q}$, $\:\overline{F}$, $\:\overline{K}$, $\:\overline{A}={A}_{1}+{B}_{1}K$, $\:\overline{B}={B}_{2}$, $\:\overline{{C}_{1}}={C}_{1}+{D}_{1}K$ into Eqs. (28), (29), (30) and (31) can be obtained:

33 $$\:\left[\begin{array}{ccc}{\left[{A}_{1}\overline{F}+{B}_{1}\overline{K}\right]}_{s}&\:{B}_{2}&\:\overline{F}({C}_{1}+{D}_{1}K{)}^{T}\\\:{{B}_{2}}^{T}&\:-\eta\:I&\:0\\\:{C}_{1}\overline{F}+{D}_{1}\overline{K}&\:0&\:-\eta\:I\end{array}\right]<0$$

34 $$\:\left[\begin{array}{cccc}-\overline{Q}&\:\overline{P}+j{\omega\:}_{c}\overline{Q}-\overline{F}&\:0&\:0\\\:\overline{P}-j{\omega\:}_{c}\overline{Q}-\overline{F}&\:-{\omega\:}_{1}{\omega\:}_{2}\overline{Q}+{\left[{A}_{1}\overline{F}+{B}_{1}\overline{K}\right]}_{s}&\:{B}_{2}&\:{\overline{F}}^{T}{{C}_{1}}^{T}+{\overline{K}}^{T}{{D}_{1}}^{T}\\\:0&\:{{B}_{2}}^{T}&\:-{\gamma\:}^{2}I&\:0\\\:0&\:{C}_{1}\overline{F}+{D}_{1}\overline{K}&\:0&\:-I\end{array}\right]<0$$

35 $$\:\left[\begin{array}{cc}-I&\:\sqrt{\rho\:}\:\overline{K}\\\:*&\:-{u}_{max}^{2}\overline{F}\end{array}\right]\le\:0$$

36 $$\:\left[\begin{array}{cc}-I&\:\sqrt{\rho\:}{\left\{\overline{{C}_{2}}\right\}}_{i}\overline{F}\\\:*&\:-\overline{F}\end{array}\right]<0,i=\text{1,2}$$

Then the transfer function of the system satisfies Eq. (26), then the closed-loop system is asymptotically stable.

The closed-loop system (24) needs to satisfy that there is a state feedback controller $\:u\left(t\right)=Kx\left(t\right)$ in the finite frequency domain such that the system:

- Asymptotically stable at *w*(*t*) = 0.
- The transfer function $\:{T}_{z\omega\:}\left(j\omega\:\right)$ from parameter variation and external disturbance to output satisfies $\left\| {T_{{zw}} \left( {j\omega \:} \right)} \right\|_{{\infty \:}} = \mathop {sup}\limits_{{\omega _{1} < \omega < \omega _{2} }} \left\| {\frac{{z_{{\infty \:}} \left( {j\omega \:} \right)}}{{w\left( {j\omega \:} \right)}}} \right\|_{{\infty \:}} < \gamma$ in finite frequency domain.
- The constraint condition $\:{max}\left|{\left\{y\left(t\right)\right\}}_{k}\right|<1$ is guaranteed when the external disturbance energy is less than $\:{w}_{\text{max}}=\sqrt{\rho\:/\eta\:}$.

Inequality (34) contains complex variable elements, which cannot be solved directly by LMI toolbox. It also needs to be transformed into the form of real linear matrix inequalities, that is:

37 $$\:{S}_{1}+j{S}_{2}<0$$

equivalent to

38 $$\:\left[\begin{array}{cc}{S}_{1}&\:{S}_{2}\\\:-{S}_{2}&\:{S}_{1}\end{array}\right]<0$$

where, definition

$$\:{S}_{1}=\left[\begin{array}{cccc}-\overline{Q}&\:\overline{P}-\overline{F}&\:0&\:0\\\:\overline{P}-\overline{F}&\:-{\omega\:}_{1}{\omega\:}_{2}\overline{Q}+{\left[{A}_{1}\overline{F}+{B}_{1}\overline{K}\right]}_{s}&\:{B}_{2}&\:{\overline{F}}^{T}{{C}_{1}}^{T}+{\overline{K}}^{T}{{D}_{1}}^{T}\\\:0&\:{{B}_{2}}^{T}&\:-{\gamma\:}^{2}I&\:0\\\:0&\:{C}_{1}\overline{F}+{D}_{1}\overline{K}&\:0&\:-I\end{array}\right],\:{S}_{2}=\left[\begin{array}{cccc}0&\:{\omega\:}_{c}\overline{Q}&\:0&\:0\\\:-{\omega\:}_{c}\overline{Q}&\:0&\:0&\:0\\\:0&\:0&\:0&\:0\\\:0&\:0&\:0&\:0\end{array}\right].$$

In summary, if the inequality (38) has a feasible solution, then the gain *K* in controller $\:u\left(t\right)=Kx\left(t\right)$ can be solved by the following:

39 $$\:K=\overline{K}\:{\overline{F}}^{-1}$$

### Design of LPV-H_∞_ controller

**Fig. 14:**
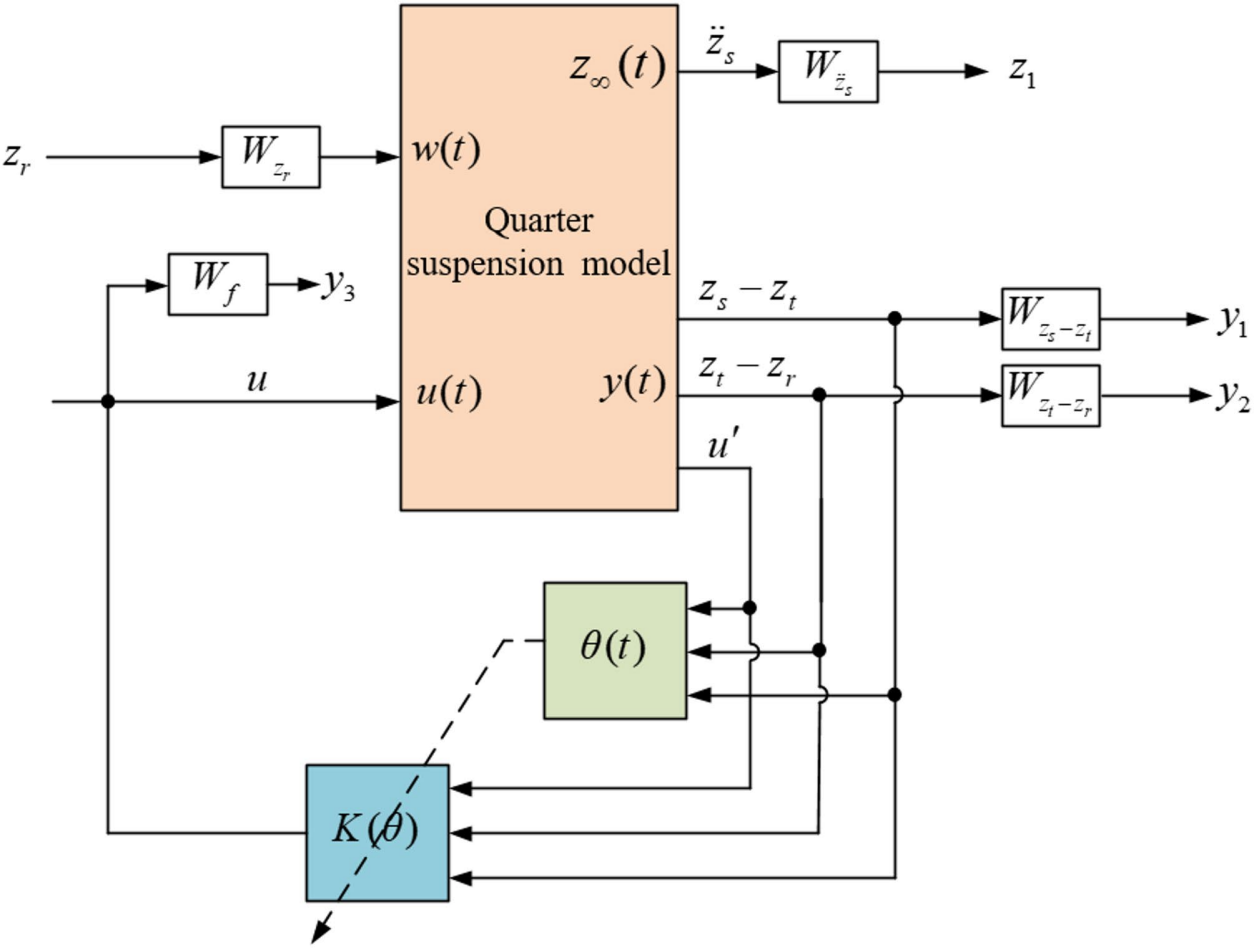
LPV-H_∞_ controller structure.

Under the requirement of high velocity suspension movement and ensuring robustness, the LPV-H_∞_ dynamic output feedback controller $\:\:u\left(t\right)=K\left(\theta\:\right)x\left(t\right)$ needs to be designed, and the structure is shown in Fig. 14.

When applying the quadratic Lyapunov function to the LPV- H_∞_ system for stability analysis and controller design, it is required that there exists a common Lyapunov function on all possible variable parameter trajectories. Due to the continuity of the variable parameters, this means that an infinite number of LMIs need to be solved to obtain the function matrix, which is unrealistic. If the system can be represented as a polytope structure, According to the properties of convex sets, only the stability analysis of the finite vertex system of the convex hull needs to be carried out to ensure the stability of the entire system. The vertex properties of the polytope LPV- H_∞_ system are as follows.

By switching the LPV system to ($\:\theta\:\in\:{R}^{l}$), the following continuous linear system in the form of LPV state space can be obtained:

40 $$\:\left\{\begin{array}{c}\dot{x}\left(t\right)={A}_{1}\left(\theta\:\right)x\left(t\right)+{B}_{1}\left(\theta\:\right)u\left(t\right)+{B}_{2}\left(\theta\:\right)w\left(t\right)\\\:{z}_{\infty\:}\left(t\right)={C}_{1}\left(\theta\:\right)x\left(t\right)+{D}_{1}\left(\theta\:\right)u\left(t\right)\\\:y\left(t\right)={C}_{2}\left(\theta\:\right)x\left(t\right)+{D}_{2}\left(\theta\:\right)u\left(t\right)\\\:\sigma\:\left(t\right)=C\left(\theta\:\right)x\left(t\right)\end{array}\right.$$

where,

$$\:\left[\begin{array}{ccc}{A}_{1}&\:{B}_{1}&\:{B}_{2}\\\:{C}_{1}&\:{D}_{1}&\:0\\\:{C}_{2}&\:{D}_{2}&\:0\end{array}\right]\in\:\varTheta\:,\:Co\left\{\left[\begin{array}{ccc}{A}_{1i}&\:{B}_{1i}&\:{B}_{2i}\\\:{C}_{1i}&\:{D}_{1i}&\:0\\\:{C}_{2i}&\:{D}_{2i}&\:0\end{array}\right],i=\text{1,2},\cdots\:,l\right\}$$

State vector is $\:x\left(t\right)\in\:{R}^{{n}_{x}}$; input vector is $\:u\left(t\right)\in\:{R}^{{n}_{u}}$; interference input is $\:w\left(t\right)\in\:{R}^{{n}_{\omega\:}}$; the system output is $\:y\left(t\right)\in\:{R}^{{n}_{y}}$.$\:\:{z}_{\infty\:}\left(t\right)\in\:{R}^{{n}_{z}}$ is the evaluation output under the performance index H_∞_. Suspension expansion velocity $\:v\left(t\right)$ represents the measurement $\Theta$output, $\:C$ can reflect a measurable amount of state, Θ is a convex polyhedron.

It can be seen from Eq. (23) that the system matrix depends on the sprung mass. Assuming that each coefficient matrix is an LPV matrix of appropriate dimension, then the affine functions $\:{A}_{1}\left(\theta\:\right)$, $\:{B}_{1}\left(\theta\:\right)$, $\:{B}_{2}\left(\theta\:\right)$, $\:{C}_{1}\left(\theta\:\right)$, $\:{C}_{2}\left(\theta\:\right)$, $\:{D}_{1}\left(\theta\:\right)$, depend on the changing parameter vector $$\:\theta\:=\left[\begin{array}{cccc}{\theta\:}_{1}&\:{\theta\:}_{2}&\:\cdots\:&\:{\theta\:}_{l}\end{array}\right]$$ $\:(\theta\:=1/{m}_{1}\in\:{R}^{l}=(1/\text{387,1}/287\left)\right)$ and the external disturbance $$\:\omega\:\left(t\right)\in\:l\left.\begin{array}{cc}[0&\:+\infty\:\end{array}\right)$$.

The coefficient matrices of the above LPV model satisfy the following polytope structure :

41 $$\:\left({A}_{1}\right(\theta\:),{B}_{1}(\theta\:),{B}_{2}(\theta\:),{C}_{1}(\theta\:),{D}_{1}(\theta\:),{C}_{2}(\theta\:),{D}_{2}(\theta\:\left)\right)={\sum\:}_{i=1}^{N}{\alpha\:}_{i}({A}_{1i},{B}_{1i},{B}_{2i},{C}_{1i},{D}_{1i},{C}_{2i},{D}_{2i})$$

where, $\:{\alpha\:}_{i}>0$, $\:{\sum\:}_{i=1}^{N}{\alpha\:}_{i}=1$. $\:{A}_{1i}$, $\:{B}_{1i}$, $\:{B}_{2i}$, $\:{C}_{1i}$, $\:{C}_{2i}$, $\:{D}_{1i}$, $\:{D}_{2i}$ are the set of $\:R\:$ vertices of all convex polyhedron, *N* is the number of vertices. Where the vertex $\:{\theta\:}_{i}\left(t\right)$ can be determined by the combination of elements $\:{\overline{\theta\:}}_{i}$ and $\:{\underset{\_}{\theta\:}}_{i}$ of the upper and lower bounds of the parameter vector, both of which depend on the vector $\:S\in\:{R}^{k}$.

42 $$\:S\left(\theta\:\right)=\left[\begin{array}{ccc}{A}_{1}\left(\theta\:\right)&\:{B}_{1}\left(\theta\:\right)&\:{B}_{2}\left(\theta\:\right)\\\:{C}_{1}\left(\theta\:\right)&\:{D}_{1}\left(\theta\:\right)&\:0\\\:{C}_{2}\left(\theta\:\right)&\:{D}_{2}\left(\theta\:\right)&\:0\end{array}\right]={\sum\:}_{i=1}^{l}{\theta\:}_{i}{S}_{i}={\theta\:}_{1}{S}_{1}+{\theta\:}_{2}{S}_{2}+\cdots\:+{\theta\:}_{l}{S}_{l}$$

The nonlinear H_∞_ control actually stabilizes the closed-loop system and minimizes the H_∞_ norm of the external disturbance *w* to the output $\:{z}_{\infty\:}$. For the polytope LPV model, the dynamic output feedback gain is assumed to be:

43 $$\:K\left(\theta\:\right):=\left\{\begin{array}{c}{\dot{x}}_{K}\left(t\right)={A}_{K}\left(\theta\:\right){x}_{K}\left(t\right)+{B}_{K}\left(\theta\:\right)\sigma\:\left(t\right)\\\:u\left(t\right)={C}_{K}\left(\theta\:\right){x}_{K}\left(t\right)+{D}_{K}\left(\theta\:\right)\sigma\:\left(t\right)\end{array}\right.$$

The combinatorics Eq. (40) and Eq. (43), define $$\:\xi\:\left(t\right)={\left[\begin{array}{cc}x\left(t\right)&\:{x}_{K}\left(t\right)\end{array}\right]}^{T}$$, The closed-loop system constituting the suspension system is:

44 $$\:\left\{\begin{array}{c}\dot{\xi\:}\left(t\right)={A}_{cl}\left(\theta\:\right)\xi\:\left(t\right)+{B}_{cl}\left(\theta\:\right)w\left(t\right)\\\:{z}_{\infty\:}\left(t\right)={C}_{1,cl}\left(\theta\:\right)\xi\:\left(t\right)\\\:y\left(t\right)={C}_{2,cl}\left(\theta\:\right)\xi\:\left(t\right)\end{array}\right.$$

The closed-loop transfer function of $\:w$ on $\:{z}_{\infty\:}$ is defined as:

45 $$\:G_{\mathrm{zw}}(j\omega) = C_{1,cl}(\theta)(j\omega I - A_{cl}(\theta))^{-1} B_{cl}(\theta)$$

where,

$$\:\left\{\begin{array}{c}{A}_{cl}\left(\theta\:\right)=\left[\begin{array}{cc}{A}_{1}\left(\theta\:\right)+{B}_{1}\left(\theta\:\right){D}_{K}\left(\theta\:\right)C\left(\theta\:\right)&\:{B}_{1}\left(\theta\:\right){C}_{K}\left(\theta\:\right)\\\:{B}_{K}\left(\theta\:\right)C\left(\theta\:\right)&\:{A}_{K}\left(\theta\:\right)\end{array}\right]\\\:{B}_{cl}\left(\theta\:\right)=\left[\begin{array}{c}{B}_{2}\left(\theta\:\right)\\\:0\end{array}\right]\\\:{C}_{1,cl}\left(\theta\:\right)=\left[\begin{array}{cc}{C}_{1}\left(\theta\:\right)+{D}_{1}\left(\theta\:\right){D}_{K}\left(\theta\:\right)C\left(\theta\:\right)&\:{D}_{1}\left(\theta\:\right){C}_{K}\left(\theta\:\right)\end{array}\right]\\\:{C}_{2,cl}\left(\theta\:\right)=\left[\begin{array}{cc}{C}_{2}\left(\theta\:\right)+{D}_{2}\left(\theta\:\right){D}_{K}\left(\theta\:\right)C\left(\theta\:\right)&\:{D}_{2}\left(\theta\:\right){C}_{K}\left(\theta\:\right)\end{array}\right]\\\:{D}_{cl}\left(\theta\:\right)={D}_{K}\left(\theta\:\right)\end{array}\right.$$.

For a given constant $\:\gamma\:$, if there exists a Lyapunov function $\:V\left(x\right)={x}^{T}Px$ in the closed-loop uncertain system, there exists a symmetric positive definite matrix $\:{P}_{s}>0$, $\:{F}_{s}>0$, which satisfies the condition of negative definite matrix such that:

46 $$\:{A}_{cl}(\theta\:{)}^{T}{F}_{s}+{F}_{s}{A}_{cl}(\theta\:)<0$$

Then the LMI set is established:

47 $$\:\left[\begin{array}{ccc}{{A}_{cl}}^{T}\left(\theta\:\right){F}_{s}+{F}_{s}{A}_{cl}\left(\theta\:\right)&\:{F}_{s}{B}_{cl}\left(\theta\:\right)&\:{{C}_{1,cl}}^{T}\left(\theta\:\right)\\\:\text{*}&\:-\eta\:I&\:0\\\:\text{*}&\:\text{*}&\:-\eta\:I\end{array}\right]<0$$

48 $$\:\left[\begin{array}{cc}-I&\:\sqrt{\rho\:}{\left\{{C}_{2}\right\}}_{i}\\\:\text{*}&\:-{F}_{s}\end{array}\right]<0,i=\text{1,2}$$

Decompose the positive definite matrix $\:{F}_{s}$ into

49 $$\:{F}_{s}=\left[\begin{array}{cc}Y&\:N\\\:{N}^{T}&\:\#\end{array}\right],\:{F}_{s}^{-1}=\left[\begin{array}{cc}X&\:M\\\:{M}^{T}&\:\#\end{array}\right]$$

Where, ‘#’ represents any matrix, which does not affect the proof and derivation. Suppose both *M* and *N* are invertible matrices, define

50 $$\:{F}_{s}{\varOmega\:}_{1}={\varOmega\:}_{2}$$

Where,

$$\:{\varOmega\:}_{1}=\left[\begin{array}{cc}X&\:I\\\:{M}^{T}&\:0\end{array}\right],\:{\varOmega\:}_{2}=\left[\begin{array}{cc}I&\:Y\\\:0&\:{N}^{T}\end{array}\right]$$

According to Eq. (22), it can be obtained

51 $$\:M{N}^{T}=I-XY$$

The controller coefficient deformation is as follows

52 $$\:\left\{\begin{array}{c}\begin{array}{c}{\widehat{A}}_{cl}\left(\theta\:\right)=N{A}_{K}\left(\theta\:\right){M}^{T}+N{B}_{K}\left(\theta\:\right){C}_{2}\left(\theta\:\right)X+Y{B}_{1}\left(\theta\:\right){C}_{K}\left(\theta\:\right){M}^{T}\\\:\:\:\:\:\:\:\:\:\:\:\:+Y({A}_{1}\left(\theta\:\right)+{B}_{1}\left(\theta\:\right){D}_{K}\left(\theta\:\right){C}_{2}\left(\theta\:\right))X\end{array}\\\:{\widehat{B}}_{cl}\left(\theta\:\right)=N{B}_{K}\left(\theta\:\right)+Y{B}_{1}\left(\theta\:\right){D}_{K}\left(\theta\:\right)\\\:{\widehat{C}}_{1,cl}\left(\theta\:\right)={C}_{K}\left(\theta\:\right){M}^{T}+{D}_{K}\left(\theta\:\right){C}_{2}\left(\theta\:\right)X\\\:{\widehat{D}}_{cl}\left(\theta\:\right)={D}_{K}\left(\theta\:\right)\end{array}\right.$$

Define

53 $$\:\left\{\begin{array}{c}{J}_{s1}=\text{diag}\left\{{\varOmega\:}_{1},I,I\right\}\\\:{J}_{s2}=\text{diag}\left\{I,{\varOmega\:}_{1}\right\}\end{array}\right.$$

The matrix $\:{J}_{s1}$ and $\:{J}_{s2}$ are used for congruent transformation of Eqs. (47) and (48) by left multiplying them and right multiplying their transposes respectively.

The following linear matrix inequality is obtained

$$\:\left[\begin{array}{cccc}{\psi\:}_{11}&\:{\psi\:}_{12}&\:{\psi\:}_{13}&\:{\psi\:}_{14}\\\:*&\:{\psi\:}_{22}&\:{\psi\:}_{23}&\:{\psi\:}_{24}\\\:*&\:*&\:-{\gamma\:}^{2}I&\:0\\\:*&\:*&\:*&\:-I\end{array}\right]<0$$

54 $$\:\left[\begin{array}{cc}X&\:I\\\:I&\:Y\end{array}\right]>0$$

where,

$$\:\left\{\begin{gathered}\psi_{11} = A_1(\theta)X + XA_1^T(\theta) + B_1(\theta)\hat{C}_{1,cl}(\theta) + \left(B_1(\theta)\hat{C}_{1,cl}(\theta)\right)^T \\\psi_{12} = \hat{A}_{cl}^T(\theta) + A_1(\theta) \\\psi_{13} = B_2(\theta) \\\psi_{14} = \left(C_1(\theta)M + D_1(\theta)\hat{C}_{1,cl}(\theta)\right)^T \\\psi_{22} = A_1^T(\theta)Y + YA_1(\theta) + \hat{B}_{cl}(\theta)C_2(\theta) + \left(\hat{B}_{cl}(\theta)C_2(\theta)\right)^T \\\psi_{23} = YB_2(\theta) \\\psi_{24} = C_1^T(\theta)\end{gathered}\right.$$

Then the above LPV closed-loop system (44) has a dynamic output feedback controller $\:u\left(t\right)=K\left(\theta\:\right)x\left(t\right)$ such that the system:

- Asymptotically stable at *w*(*t*) = 0.
- The transfer function $\:{T}_{z\omega\:}\left(j\omega\:\right)$ from parameter variation and external disturbance to output satisfies $\mathop {sup}\limits_{{\omega \:_{1} < \omega < \omega \:_{2} }} \left\| {G_{{z\omega \:}} \left( {j\omega \:} \right)} \right\|_{{\infty \:}} < \gamma \:$ in finite frequency domain.
- The constraint condition $\:{max}\left|{\left\{y\left(t\right)\right\}}_{k}\right|<1$ is guaranteed when the external disturbance energy is less than $\:{w}_{\text{max}}=\sqrt{\rho\:/\eta\:}$.

If $$\:X$$, $$\:Y$$, $$\:\overset{\lower0.5em\hbox{$\smash{\scriptscriptstyle\frown}$}}{A} _{{cl}} \left( {\theta \:} \right)$$, $$\:{\widehat{B}}_{cl}\left(\theta\:\right)$$, $$\:{\widehat{C}}_{1,cl}\left(\theta\:\right)$$ and $$\:{\widehat{D}}_{cl}\left(\theta\:\right)$$ can be solved$$\:\:M$$ and$$\:\:N$$ by singular value decomposition of a matrix $$\:M{N}^{T}=I-XY$$, define a controller

55 $$\:\left\{\begin{gathered}A_K = N^{-1}\big[\hat{A}_{cl}(\theta) - YA_1(\theta)X - YB_1(\theta)D_K(\theta)C_2(\theta)X - NB_K(\theta)C_2(\theta)X \\\quad\quad - YB_1(\theta)C_K(\theta)M^T\big](M^T)^{-1} \\B_K(\theta) = N^{-1}\big(\hat{B}_{cl}(\theta) - YB_1(\theta)D_K(\theta)\big) \\C_K(\theta) = \big(\hat{C}_{1,cl}(\theta) - D_K(\theta)C_2(\theta)X\big)(M^T)^{-1} \\D_K(\theta) = \hat{D}_{cl}(\theta)\end{gathered}\right.$$

By solving Eq. (55), the dynamic output feedback gain $\:K\left(\theta\:\right)$ of the controller $\:u\left(t\right)=K\left(\theta\:\right)x\left(t\right)$ can be obtained.

### Simulation and analysis

Considering that the more sensitive frequency range of the human body is $\:4\sim8\text{Hz}$, therefore, the vibration control of the suspension system should pay more attention to the control effect within $\:4\sim8\text{Hz}$. Given scalar value $\:{\omega\:}_{1}=4\text{Hz}$, $\:{\omega\:}_{2}=8\text{Hz}$, $\:\rho\:=1$, $\:\eta\:=8000$, maximum dynamic travel of suspension is $\:{z}_{max}=0.1\text{m}$, maximum output force of MR damper is $\:{u}_{max}=1800\text{N}$.

By solving the Eq. (38), the optimal $\:{\gamma\:}_{min}=6.874$ is obtained. Using the MATLAB standard LMI toolbox, the corresponding state feedback controller and dynamic feedback controller are solved, and the closed-loop system is analyzed. Table 4 shows the basic parameters of the suspension.

**Table 4:** Suspension system parameters.

Parameters	Symbol	Values	Unit
Sprung mass	*m*_s_	287	kg
Unsprung mass	*m*_t_	40	kg
Air spring stiffness	*k*_s_	1.1 × 10^4^	N·m^− 1^
Tire stiffness	*k*_t_	1.8 × 10^5^	N·m^− 1^
Zero-field damping coefficient	*c*_0_	1500	N·s·m^− 1^
Effective action area of air spring	*A*_*e*_	0.0381	m^2^
Initial volume of air spring	*V*_0_	0.0078	m^3^
Initial pressure of air spring	*P*_0_	0.382	MPa

### Speed bump road excitation

**Fig. 15:**
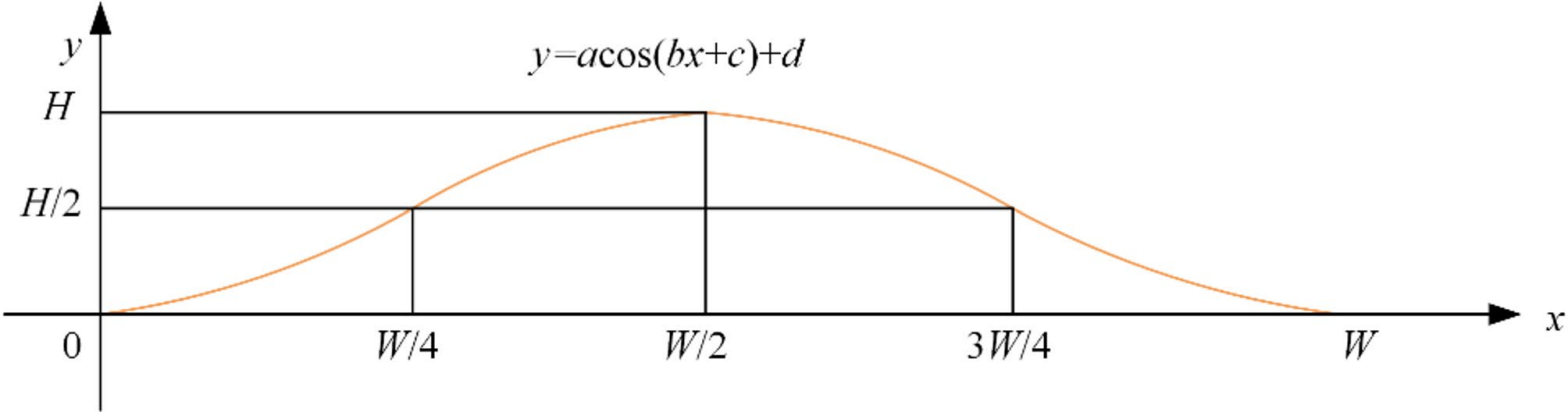
Modeling of the speed bump.

In order to verify the vibration reduction effect of the suspension under the action of the speed bump road surface, this paper establishes the speed bump road surface model under the cosine section as shown in Fig. 15, and its expression is Eq. (51).

51 $$\:{z}_{r}\left(t\right)=\left\{\begin{array}{cc}\frac{H}{2}\left[1-{cos}\left(\frac{2\pi\:v}{W}t\right)\right]&\:\frac{W}{v}\le\:t\le\:\frac{2W}{v}\\\:0&\:\text{others}\end{array}\right.$$

where, $\:{z}_{r}\:$is the signal of the speed bump road surface; The speed bump height is $\:H=60\text{mm}$; The width of the speed bump is $\:W=0.08m$; The velocity of the vehicle is $\:v=5\text{km/h}$.

**Fig. 16:**
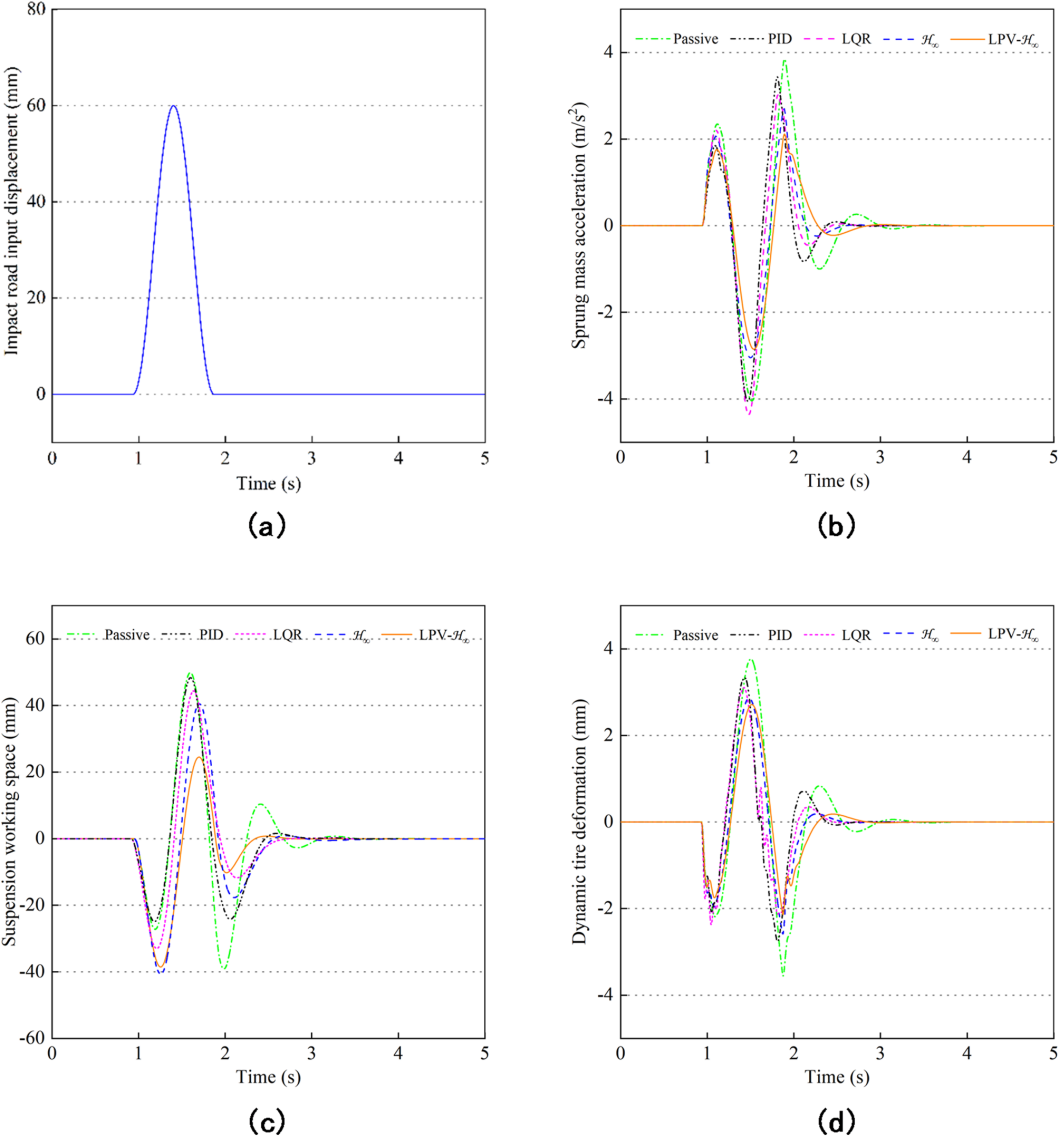
Simulation curves diagram under speed bump pavement. (**a**) Impact road input. (**b**) Sprung mass acceleration. (**c**) Suspension working space. (**d**) Dynamic tire deformation.

Figure 16 shows the vibration effects of the five controls on the road excitation of the speed bump. Figure 16(a) shows the speed bump pavement in about one second. Figure 16(b-d) shows the SMA, SWS and DTD simulation results of MR semi-active suspension with passive control, PID control, LQR control, H_∞_ control, and LPV-H_∞_ control under speed bump road excitation respectively. The curves described in the results show that compared with others, the use of LPV-H_∞_ control in the semi-active suspension makes the root mean square value significantly reduced, indicating that when the vehicle passes through harsh road conditions such as shocks and speed bumps, the vibration energy transmitted to the body is greatly suppressed, and the ride comfort of the vehicle is improved.

Table 5 shows the RMS and improvement rate of the suspension performance under the speed bump road. Compared with the passive control, the PID control improves the suspension performance by 6.80%, 2.93% and 8.41%, respectively; The LQR control improves the suspension performance by 10.79%, 4.63% and 12.33%, respectively; The H_∞_ control improves the suspension performance by 27.24%, 15.16% and 25.92%, respectively; The LPV-H_∞_ control improves the suspension performance by 33.16%, 32.31% and 30.40% respectively, and LPV-H_∞_ is further optimized by 5.92%, 17.15% and 4.48% compared with H_∞_. It can be known from this that the semi-active suspension system controlled by LPV-H_∞_ can reduce the influence of speed bumps on the road surface and improve the stability and comfort of the vehicle.

**Table 5:** RMS and improvement rate of suspension performance under speed bump pavement.

Control strategy	SMA$\:\left(\text{m/}{\text{s}}^{2}\right)$		SWS$\:(\text{mm)}$		DTD$\:(\text{mm)}$	
	RMS	Improve rate	RMS	Improve rate	RMS	Improve rate
Passive	0.6281	0%	14.8192	0%	1.1505	0%
PID	0.5854	6.80%	14.3853	2.93%	1.0538	8.41%
LQR	0.5603	10.79%	14.1326	4.63%	1.0086	12.33%
H_∞_	0.4570	27.24%	12.5720	15.16%	0.8523	25.92%
LPV-H_∞_	0.4198	33.16%	10.0305	32.31%	0.8007	30.40%

### Random road excitation

In order to verify the effectiveness of the system control, this paper takes the C-level road excitation as the road input for further analysis. The time-domain random road excitation model is usually established by white noise, which can be expressed as:

$$\:\dot{z}_{r} \left( t \right) = - 2\pi \:f_{{\min }} z_{r} \left( t \right) + 2\pi \:n_{0} \sqrt {G_{{xr}} \left( {n_{0} } \right)v} w\left( t \right)$$

where, $\:{\dot{z}}_{r}\left(t\right)$ is the time domain representation of road excitation; $\:{f}_{min}$ is the cut-off frequency under time; $\:{n}_{0}$ is the reference spatial frequency; $\:{G}_{xr}\left({n}_{0}\right)$ is the road roughness coefficient, $\:v$ is the velocity of the vehicle; $\:w\left(t\right)$ is standard Gaussian white noise.

**Fig. 17:**
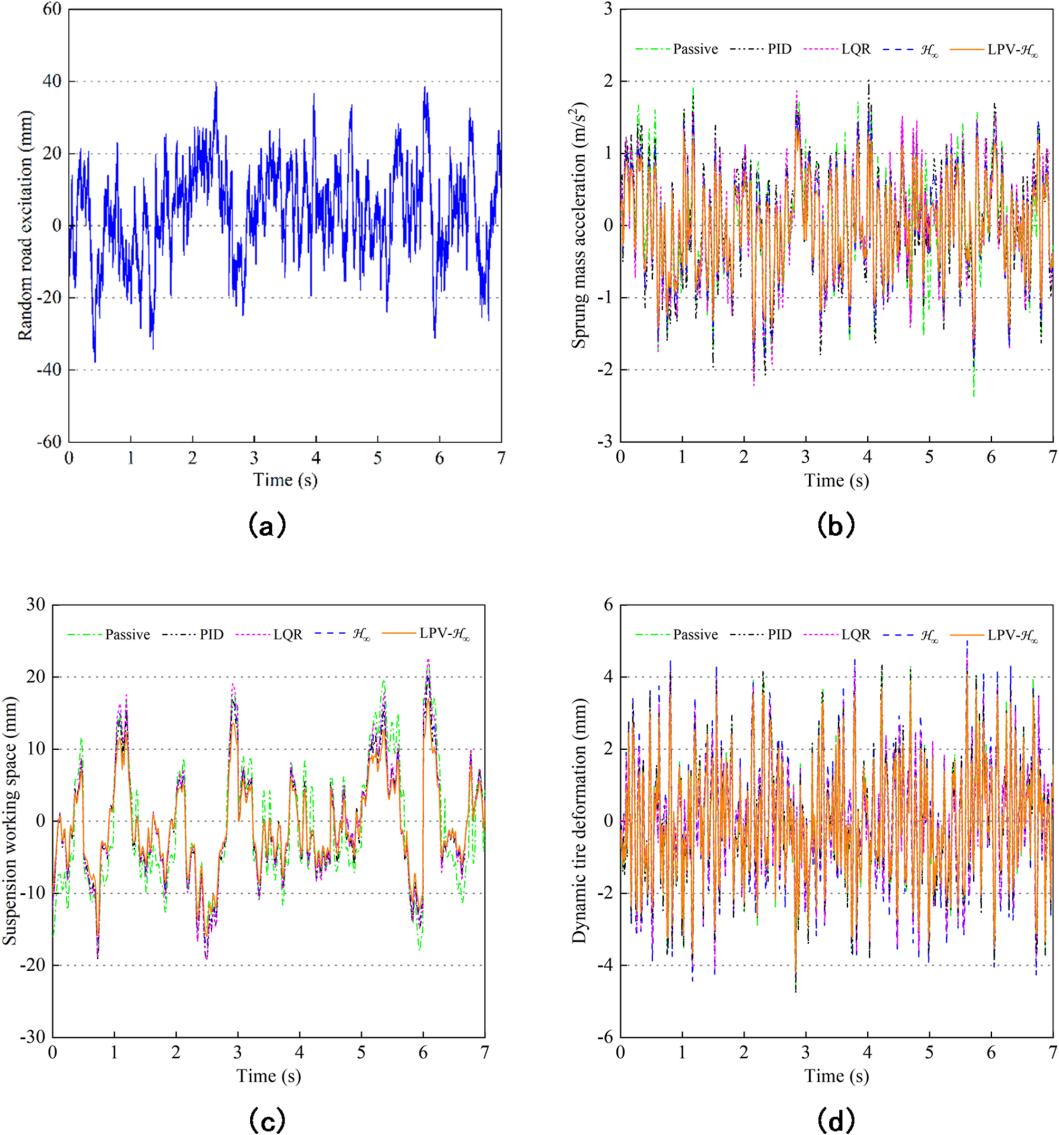
Simulation curves under random road excitation. (**a**) Random road input. (**b**) Sprung mass acceleration. (**c**) Suspension working space. (**d**) Dynamic tire deformation.

The random road excitation model was built in MATLAB / Simulink, and the sampling period was 0.001s. In order to ensure the comfort requirement, the cut-off frequency under time was $\:{f}_{min}\text{=0.33Hz}$, the roughness coefficient is $\:{G}_{xr}\left({n}_{0}\right)=256\times\:1{0}^{-6}{m}^{3}$, the reference spatial frequency $\:{n}_{0}=0.1{m}^{-1}$ under C-level, and the velocity is *v=*36 km/h. Figure 17 shows the time-domain simulation results under random road excitation. The random road displacement input curve is shown in Fig. 17(a), and the time-domain simulation curves of SMA, SWS and DTD are shown in Fig. 17(b-d). It can be seen from the curve that the response peak of the curve of the semi-active suspension with LPV-H_∞_ control is lower than that of the semi-active suspension with other controls.

Table 6 shows the calculated RMS values of various performance indexes of the suspension system under five different control methods. The results show that the RMS values of the three performance indexes of the MR semi-active suspension under the LPV-H_∞_ control are lower than other controls. Compared with passive control, the PID control improves the suspension performance by1.64%, 20.34% and − 2.78%, respectively; The LQR control is optimized by 3.83%, 14.98% and − 1.13%, respectively; The H_∞_ control is optimized by 13.31%, 22.61% and − 7.80% respectively, while the LPV- H_∞_ control is optimized by 22.50%, 33.64% and 17.12%, respectively, and LPV-H_∞_ is further optimized by 9.19%, 11.03% and 24.92% compared with H_∞_. Therefore, the LPV-H_∞_ control has good robustness and improves the ride comfort and handling stability during vehicle driving.

**Table 6:** RMS and improvement rate of suspension performance under random road excitation.

Control strategy	SMA$\:\left(\text{m/}{\text{s}}^{2}\right)$		SWS$\:(\text{mm)}$		DTD$\:(\text{mm)}$	
	RMS	Improve rate	RMS	Improve rate	RMS	Improve rate
Passive	0.7546	0%	8.4219	0%	1.8207	0%
PID	0.7422	1.64%	6.7085	20.34%	1.8714	-2.78%
LQR	0.7257	3.83%	7.1605	14.98%	1.8412	-1.13%
H_∞_	0.6541	13.31%	6.5179	22.61%	1.9628	-7.80%
LPV-H_∞_	0.5848	22.50%	5.5884	33.64%	1.5090	17.12%

**Fig. 18:**
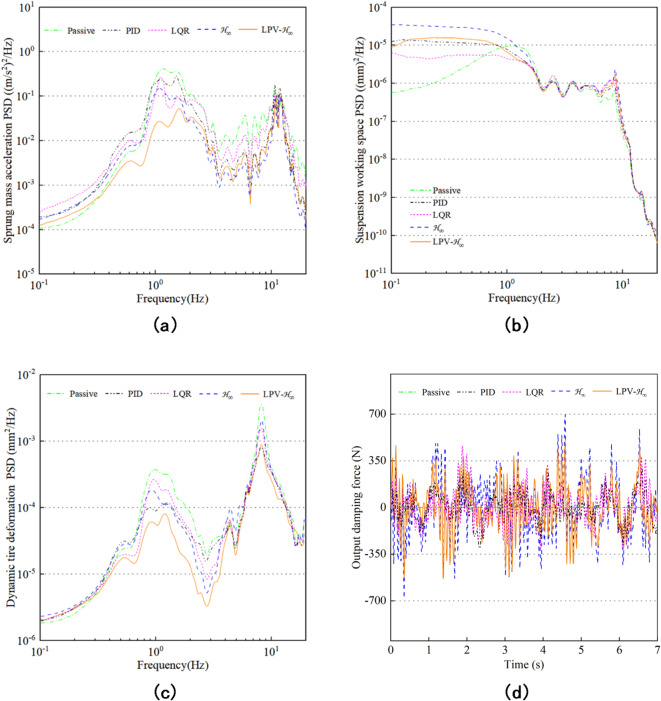
PSD of suspension system performance and output damping force. (**a**) Sprung mass acceleration PSD. (**b**) Suspension working space PSD. (**c**) Dynamic tire deformation PSD. (**d**) Output damping force.

In order to understand the performance of the suspension system more comprehensively, besides the time domain analysis, Power spectral density (PSD) analysis of the suspension system is also needed. Figure 18(a-c) shows the PSD frequency domain simulation curves of SMA, SWS, and DTD obtained by power spectral density calculation based on the results of random pavement inputs. In the low frequency resonance region, the PSD of body vertical acceleration, suspension dynamic travel and tire dynamic deformation controlled by LPV-H_∞_ are significantly improved. In the high frequency resonance region, the LPV-H_∞_ control is subject to frequency conversion interference, and the PSD of SMA and DTD almost coincides with the other controls and is superior to the passive control. Because the weighting function focuses on the sensitive frequency of human body, the high frequency performance of the system will be sacrificed, resulting in the increase of SWS. The PSD of SMA, SWS and DTD decrease significantly when LPV-H_∞_ is controlled at the human sensitive frequency 4 ~ 8 Hz. Therefore, the MR semi-active suspension system controlled by LPV-H_∞_ can reduce body resonance and improve vehicle riding comfort. Figure 18(d) shows the output force of MR damper under five controls, all within 800 N, which can be achieved.

Table 7 shows the comparison of the improve rate of the three evaluation indicators for the four controls of PID, LQR, H_∞_ and LPV-H_∞_ under different road surfaces and different sprung masses. Due to the existence of constraint conditions, the reduction of acceleration and dynamic stroke is overly required, resulting in positive optimization of the other controls for SMA and SWS, but obvious negative optimization for DTD. With the increase of sprung mass, the influence of H_∞_ control on DTD becomes more and more obvious. The negative optimization degree of PID and LQR on DTD is relatively small. The greater the road excitation, the worse the optimization effect will be. In addition, LPV-H_∞_ control demonstrates consistency and optimal performance under all conditions.

**Table 7:**
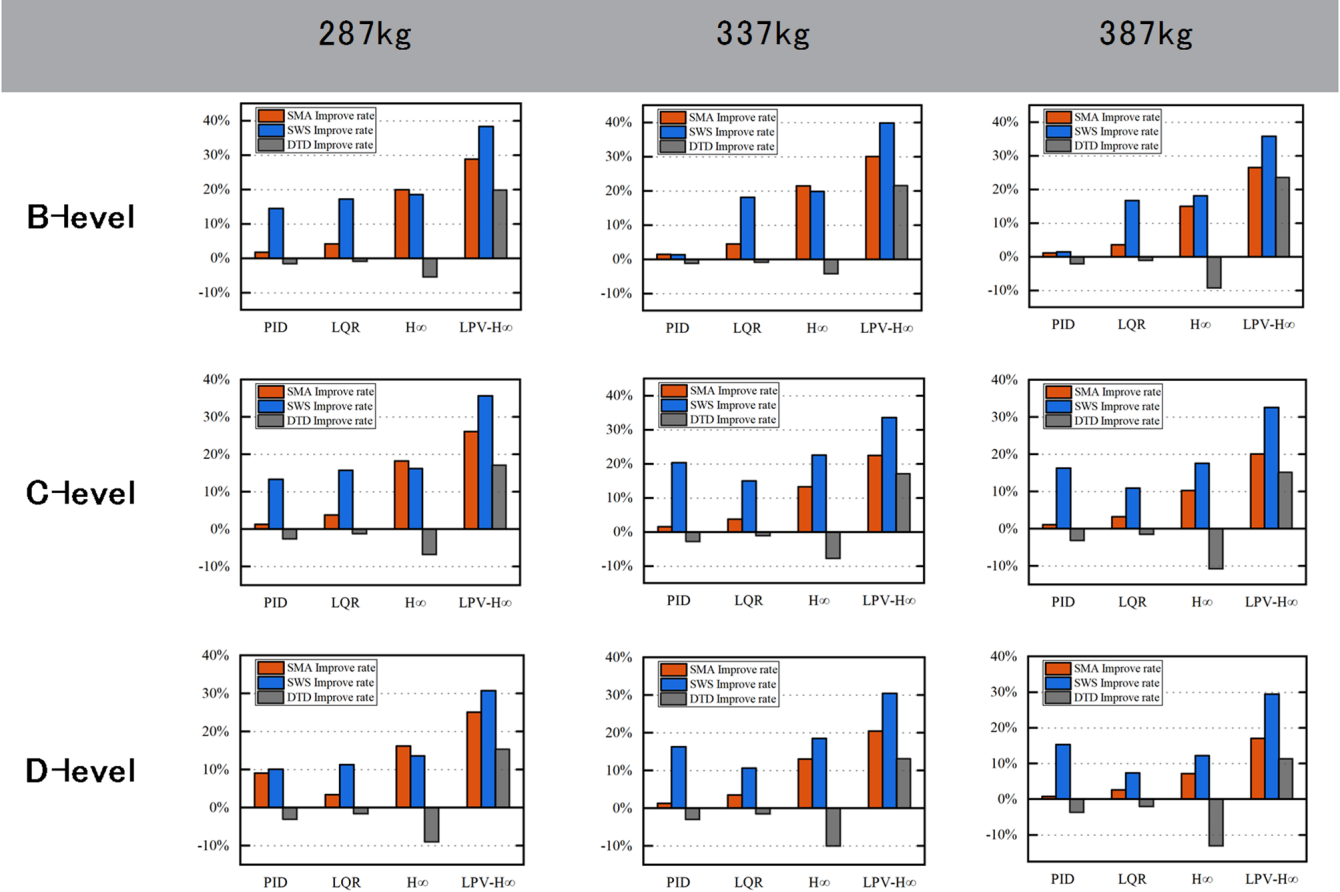
Different controls ensure smooth performance in various environments.

### Real vehicle test research

#### Test system

**Fig. 19:**
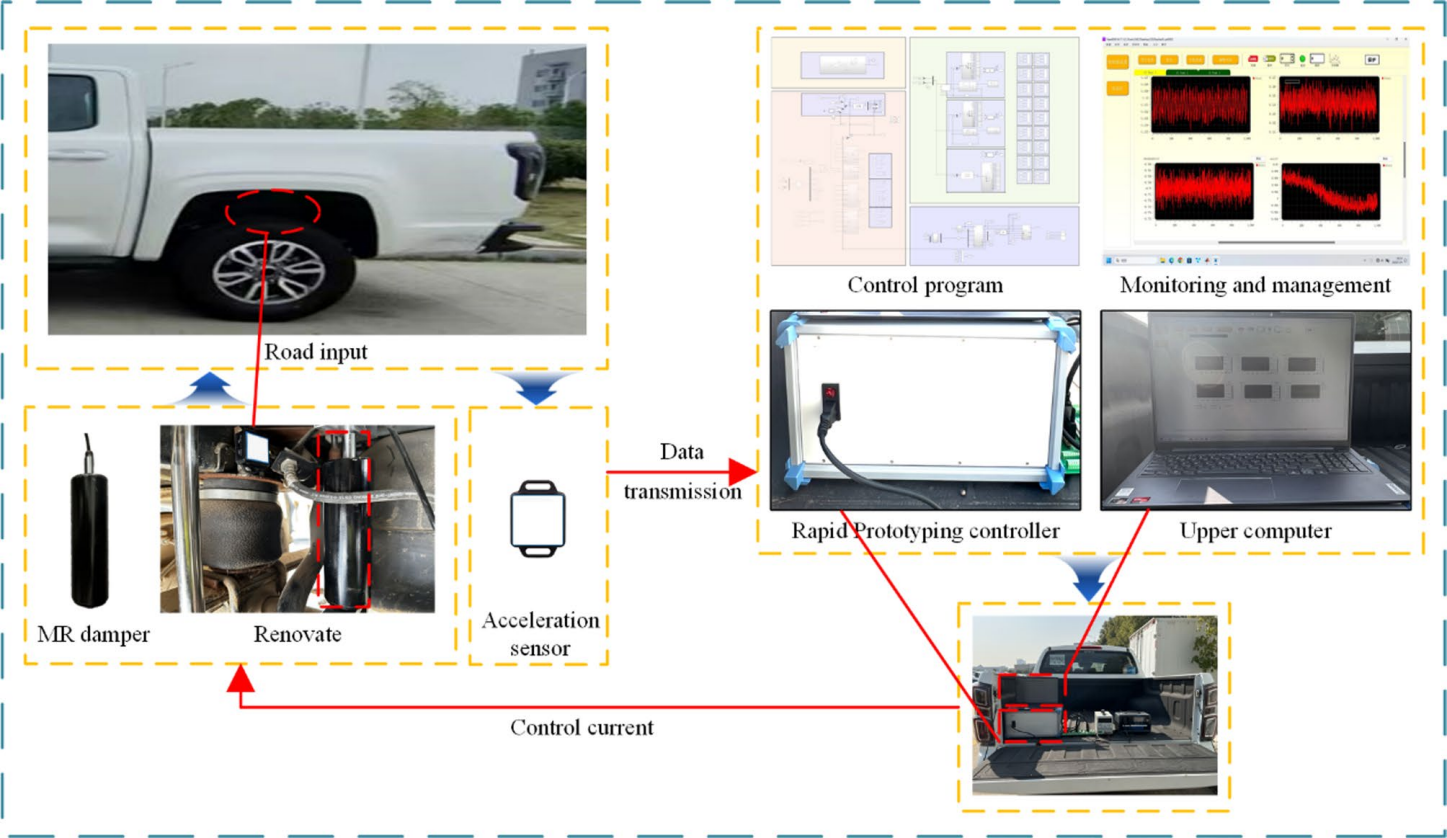
MR semi-active suspension test system with air spring.

To further verify the effectiveness of the control strategy designed in this paper, a vehicle performance test system as shown in Fig. 19 was built.

The test system replaced the original damper and metal spring of the test vehicle with MR and air spring designed by the research group. The upper computer adopted the notebook computer of the research group, which was connected to the rapid prototype controller through the WLAN port. The performance data was fed back through the acceleration sensor installed on the vehicle body and transmitted to the controller for data collection and calculation. And run the designed control strategy program to generate the control current signal of the suspension system, which is transmitted to the current drive module to output the control current, thereby achieving the control of the magnetorheological semi-active suspension system with air spring.

#### Tests on different road surfaces

**Fig. 20:**
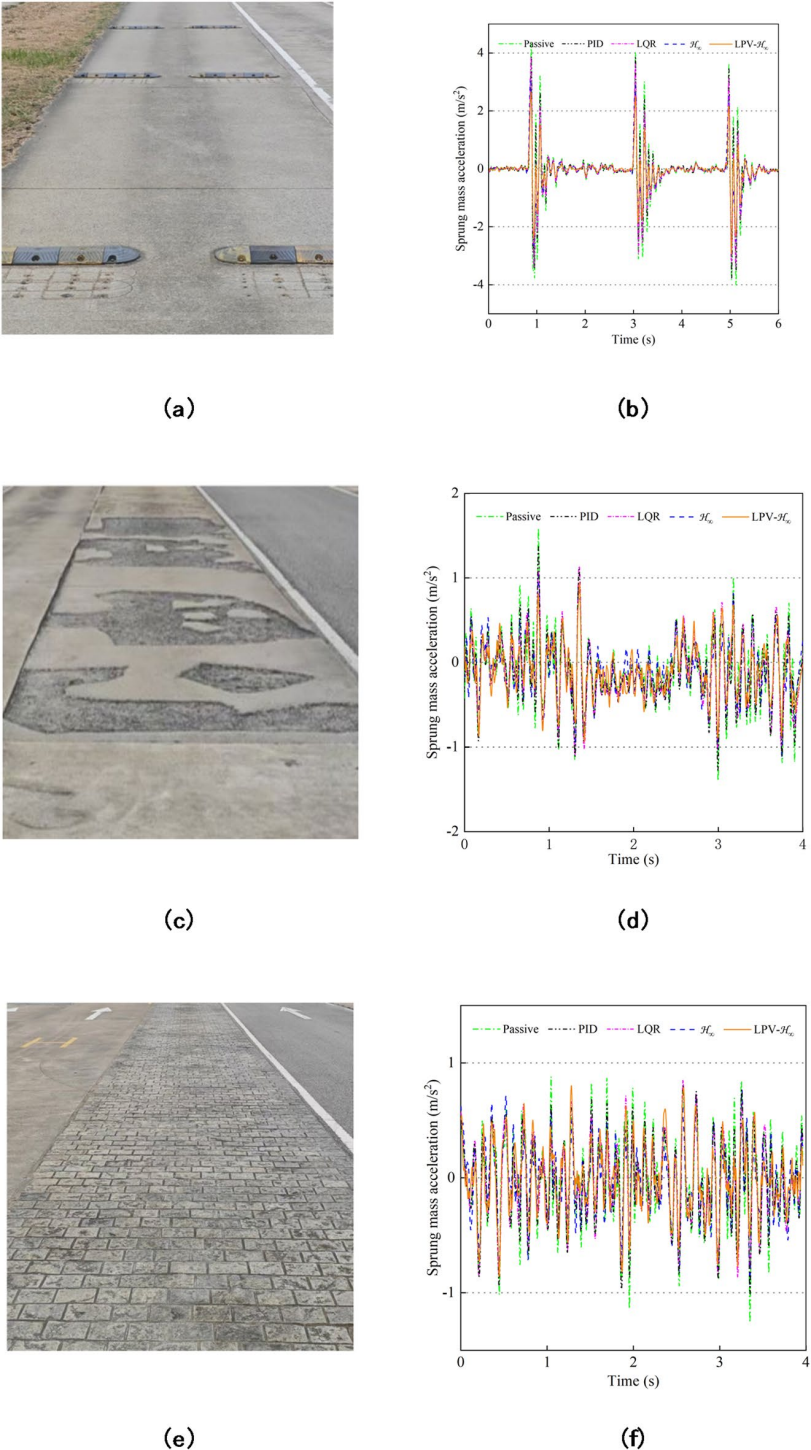
Tests on different road surfaces. (**a**) Continuous trapezoidal speed bumps road surface. (**b**) Sprung mass acceleration on continuous trapezoidal speed bumps road surface. (**c**) Bad road surface. (**d**) Sprung mass acceleration on bad road surface. (**e**) Belgian block road surface. (**f**) Sprung mass acceleration on Belgian block road surface.

Because of the ride comfort is mainly determined by the sprung mass acceleration, this paper selects the continuous trapezoidal speed bumps road surface, bad road surface and Belgian block road surface as shown in Fig. 20 as the tests road surface, and takes their accelerations as the evaluation index. The velocity is 10 km/h. By comparing the real vehicle tests results under five different controls, the effectiveness of the controller designed in this paper is verified. Among them, Fig. 20(a), (c) and (e) respectively show the continuous trapezoidal speed bumps road surface, the bad road surface, and the Belgian block road surface, and Fig. 20(b), (d) and (f) respectively show the comparison curves of the sprung mass acceleration under the three types of road surfaces.

Table 8 shows the RMS values of acceleration performance in real vehicle tests under three types of road surfaces. The test results show that, compared with passive control, PID control improves the acceleration performance under the three types of road surfaces by 7.98%, 7.83% and 12.11% respectively, LQR control optimizes by 12.29%, 13.01% and 18.38% respectively, and H_∞_ control optimizes by 24.56%, 16.84% and 19.24% respectively. LPV-H_∞_ was optimized by 31.68%, 23.80% and 22.06%, respectively. By comparing the simulation results and tests results of the five controls in Tables 6 and 8, it can be observed that under the impact road surface, the LPV-H_∞_ has a relatively obvious effect on improving vehicle performance, while on the better road surface, it will slightly improve vehicle performance. the effectiveness of the controller designed in this paper can be fully verified in the real vehicle tests, and it can improve the suspension performance of the vehicle during driving.

**Table 8:** RMS and improvement rate of different road surface tests.

Control strategy	Continuous trapezoidal speed bumps road surface		Bad road surface		Belgian block road surface	
	RMS	Improve rate	RMS	Improve rate	RMS	Improve rate
Passive	0.6897	0%	0.4595	0%	0.4319	0%
PID	0.6346	7.98%	0.4235	7.83%	0.3796	12.11%
LQR	0.6049	12.29%	0.3997	13.01%	0.3525	18.38%
H_∞_	0.5203	24.56%	0.3821	16.84%	0.3488	19.24%
LPV-H_∞_	0.4712	31.68%	0.3501	23.80%	0.3366	22.06%

## Conclusion

In this paper, the LPV-H_∞_ control has been proposed for MR semi-active suspension with air spring, and H_∞_ state feedback controller and LPV-H_∞_ dynamic output feedback controller are designed. The improved hyperbolic tangential forward model has been established by nonlinear least square method, and the simulation data is compared with the experimental data. At the same time, the inverse model of MR damper is established by using CNN to solve the current required by MR damper. In addition, the quarter MR semi-active suspension with air spring model is also established. After solving the controller through the LMI toolbox, simulations were carried out using speed bump road surface and random road surface, and real vehicle tests were carried out using continuous trapezoidal speed bumps road surface, bad road surface and Belgian road surface. The results show that the designed controller has good robustness and anti-interference ability, and the controller design is simple, which effectively improves the ride comfort and safety of the vehicle. It has practical significance in the study of semi-active suspension. However, there are still deficiencies in the research on the semi-active suspension control with the integrated installation of air spring and MR damper at present. There are still some problems in this paper that await further in-depth studies. For example, the MR damper itself has hysteresis characteristics, which makes it difficult to guarantee the accuracy of the established model. In this paper, only the improved hyperbolic tangent model and the nonlinear least squares parameter identification method are established. Subsequently, more models and methods should be adopted. Through comparative studies, the accuracy of the dynamic model of magnetorheological damper can be further improved. In this paper, when establishing the air spring model, only the stiffness characteristics of the air spring under different loading conditions were analyzed. However, in the actual control process, quantitative inflation treatment of the air spring was chosen, not only to reduce the number of variable parameters, but also to better obtain the vibration damping performance. In the subsequent research, the coupling problem of air spring and MR damper should be considered to achieve coordinated control aimed at improving vehicle ride comfort.

## Data Availability

Since this experimental data is the basis for the subsequent experimental platform, the data set generated and analyzed during the current study is not public but can be obtained from the corresponding author according to reasonable requirements.
